# Applications and efficiencies of the first cat 63K DNA array

**DOI:** 10.1038/s41598-018-25438-0

**Published:** 2018-05-04

**Authors:** Barbara Gandolfi, Hasan Alhaddad, Mona Abdi, Leslie H. Bach, Erica K. Creighton, Brian W. Davis, Jared E. Decker, Nicholas H. Dodman, Edward I. Ginns, Jennifer C. Grahn, Robert A. Grahn, Bianca Haase, Jens Haggstrom, Michael J. Hamilton, Christopher R. Helps, Jennifer D. Kurushima, Hannes Lohi, Maria Longeri, Richard Malik, Kathryn M. Meurs, Michael J. Montague, James C. Mullikin, William J. Murphy, Sara M. Nilson, Niels C. Pedersen, Carlyn B. Peterson, Clare Rusbridge, Rashid Saif, G. Diane Shelton, Wesley C. Warren, Muhammad Wasim, Leslie A. Lyons

**Affiliations:** 1 Department of Veterinary Medicine and Surgery, College of Veterinary Medicine, University of Missouri - Columbia, Columbia, MO USA; 2 Department of Biological Sciences, Kuwait University, Safat, Kuwait; 3 Department of Population Health and Reproduction, School of Veterinary Medicine, University of California – Davis, Davis, CA USA; 4 University of San Francisco, San Francisco, CA USA; 5 Department of Veterinary Integrative Biosciences, Texas A&M University, College Station, TX USA; 6 Division of Animal Sciences, University of Missouri - Columbia, Columbia, MO USA; 7 Cummings School of Veterinary Medicine, Tufts University, North Grafton, MA USA; 8 Department of Psychiatry, University of Massachusetts Medical School, Worcester, MA USA; 9 Veterinary Genetics Laboratory, School of Veterinary Medicine, University of California - Davis, Davis, CA USA; 10 Sydney School of Veterinary Science, University of Sydney, Sydney, Australia; 11 Department of Clinical Sciences, Swedish University of Agricultural Sciences, Uppsala, Sweden; 12 Department of Biochemistry, University of California – Riverside, Riverside, CA USA; 13 Langford Vets, University of Bristol, Bristol, United Kingdom; 14 Foothill College, Los Altos Hills, CA USA; 15 Department of Veterinary Biosciences, Research Programs Unit, Molecular Neurology, University of Helsinki, and The Folkhälsan Institute of Genetics, Helsinki, Finland; 16 Department of Veterinary Medicine, Università degli Studi di Milano, Milan, Italy; 17 Centre for Veterinary Education, University of Sydney, New South Wales, Australia; 18 Department of Clinical Sciences, College of Veterinary Medicine, North Carolina State University, Raleigh, NC USA; 19 Department of Neuroscience, Parelman School of Medicine, University of Pennsylvania, Philadelphia, PA USA; 20 NIH Intramural Sequencing Center, National Human Genome Research Institute, National Institutes of Health, Bethesda, MD USA; 21 Center for Companion Animal Health, School of Veterinary Medicine, University of California - Davis, Davis, CA USA; 22 School of Veterinary Medicine, Faculty of Health and Medical Sciences, University of Surrey, Guildford, Surrey United Kingdom; 23 Institute of Biotechnology, Gulab Devi Educational Complex, Lahore, Pakistan; 24 Department of Pathology, University of California, San Diego, La Jolla, CA USA; 25 McDonnell Genome Institute, Washington University School of Medicine, St Louis, MO USA; 26 Institute of Biochemistry and Biotechnology, University of Veterinary and Animal Sciences, Lahore, Pakistan

**Keywords:** Genomics, Genotype

## Abstract

The development of high throughput SNP genotyping technologies has improved the genetic dissection of simple and complex traits in many species including cats. The properties of feline 62,897 SNPs Illumina Infinium iSelect DNA array are described using a dataset of over 2,000 feline samples, the most extensive to date, representing 41 cat breeds, a random bred population, and four wild felid species. Accuracy and efficiency of the array’s genotypes and its utility in performing population-based analyses were evaluated. Average marker distance across the array was 37,741 Kb, and across the dataset, only 1% (625) of the markers exhibited poor genotyping and only 0.35% (221) showed Mendelian errors. Marker polymorphism varied across cat breeds and the average minor allele frequency (MAF) of all markers across domestic cats was 0.21. Population structure analysis confirmed a Western to Eastern structural continuum of cat breeds. Genome-wide linkage disequilibrium ranged from 50–1,500 Kb for domestic cats and 750 Kb for European wildcats (*Felis silvestris silvestris*). Array use in trait association mapping was investigated under different modes of inheritance, selection and population sizes. The efficient array design and cat genotype dataset continues to advance the understanding of cat breeds and will support monogenic health studies across feline breeds and populations.

## Introduction

Feral and owned cats are collectively referred to as “domestic cats”. Over 88 million domestic cats live in homes in the USA alone^1,2^, and are valued companions, providers of vermin control, and important biomedical models^3^. The domestic cat, *Felis silvestris catus*, represents one of the ~41 species in the family *Felidae*^4–6^ with the extant species having a common ancestor ~11 million years ago^7,8^. Previous archeological and genetic research has suggested the modern domesticated cat descends from at least one wildcat progenitor subspecies, *Felis silvestris libyca*, around 10,000 years ago^9–11^.

Agricultural development is thought to be the key event that initiated and influenced the domestication of the cat^11–13^. The availability of grains and other food sources in and around areas of human settlements resulted in substantial rodent population expansion, which in turn attracted the natural predator, the progenitor of the domestic cat, from the wildcat population. Over time, individual cats with temperaments suitable for co-habitation with human populations became isolated from the wild counterparts and evolved into the semi-domesticated cat of today. In spite of their rapid spread and isolation from the progenitor populations, domestic cats have remained remarkably similar to their felid cousins (*Felis silvestris* subsp.) in form and behavior^12,14^ and these wild populations have remained widespread across the Old World.

The establishment of cat breeds from domesticated and tamed free-roaming cat populations is a relatively recent event. Many domesticated animal species such as cattle, goats, pig, dog, and horse, were selected for traits of economic value such as meat, milk, drought tolerance, endurance, strength, protection, hunting ability, speed and metabolic efficiency from the onset of their domestication^15,16^. All these desired qualities are the products of hundreds to thousands of years of selective breeding^12,13^. However, the domestic cat breeds were selectively bred primarily for aesthetically pleasing traits such as coat color, length, and texture, most of which occurred only in the past 150 years^17,18^. Between 40 and 55 different cat breeds are currently recognized for standardized phenotypic characteristics by worldwide cat fancy associations, including the Cat Fanciers’ Association^19^, The International Cat Association^20^, the Governing Council of the Cat Fancy^21^, Federation International Feline^22^, and the World Cat Federation^23,24^. Due to inbreeding, many cat breeds harbor heritable diseases that are important biomedical models for human health (http://omia.angis.org.au/home/)^3,25^. However, owned random-bred and un-owned or semi-owned feral cats represent the overwhelming majority of cats in the world^26^.

The continued development and progress of genetic resources for humans have transformed the field of genetics and accelerated the rate of scientific discovery^27–29^. Similarly, genetic resources for the domestic cat have methodically and systematically been developed, which include somatic cell hybrid panels^30,31^, radiation hybrid maps^32–39^, genetic linkage maps^40–44^, and the sequencing of the cat genome^45–48^. Feline genome sequencing efforts to date have included: (1) a 1.9x draft sequence as a representative of the family *Felidae*^45^, (2) additional light sequencing (~1X coverage) of six individuals from several breeds and an African wild cat (*Felis silvestris cafra*) for SNPs discovery^47^, (3) high throughput sequencing of four pooled samples from each of six different domestic cat breeds, wildcats, as well as the reference cat genome^46^ and (4) a high-resolution SNP array-based linkage map that supported the assembly of Felis_catus v8.0^48^. The SNPs discovered via these sequencing efforts were used to construct an Illumina Infinium iSelect 63K DNA cat array. The produced array contains 62,897 variants that enable genome-wide case–control association studies and population-based investigations for cats rather than focusing only on pedigree analysis and candidate gene-based approaches.

Using an extensive dataset of over 2,000 cats genotyped using the feline SNP array, this study had two main objectives: firstly, to evaluate the array’s accuracy and efficiency for genome-wide genotyping that included validation tests of (1) remapping SNP physical positions to the newest cat genome assembly, (2) SNP genotyping rate, (3) SNP Mendelian inheritance, and (4) allelic variability across breeds, and secondly, to test the reliability of the array’s genotype data for population based analyses. The population-based analyses included assessments of (1) genetic diversity, (2) population structure, (3) linkage disequilibrium, and (4) association mapping.

## Results

### DNA array properties

Using the early assembly of the cat genome^45^ and the improved assembly by re-sequencing^47^ (FelCat4 (*Felis catus* 5.8)), ~10 million polymorphic variants, were submitted for design to produce a low density Illumina Infinium iSelect DNA array. SNPs that represented all known cat phenotypes and diseases at the time were submitted, as well as SNPs unique to a single assayed wildcat (*Felis cafra*)^47^. The final design (n = 62,897) included 59,469 autosomal SNPs, 2,724 X-linked SNPs **(**Supplementary Table 1), wildcat-specific SNPs (n = 4,240) and 126 SNPs representing trait-specific or disease-specific loci. A complete list of wildcat SNPs is provided in Supplementary Data File 1 and all other SNPs on the array in Supplementary Data File 2.

### Remapping array SNPs to the *Felis Catus* 8.0 cat genome assembly

The array variants were previously remapped to cat assembly 6.2^49,50^. For the 62,897 SNP positions, 62,193 (~99%) were identified in the Felis_catus_8.0 genome assembly, including 2,724X chromosome markers. The remaining 704 variants were assigned to chromosome 20, representing unknown chromosome locations (Supplementary Data File 3). Unmapped sequences were manually inspected and most had only partial alignments to the reference. The final SNP map maintained the same order as the remapping to cat assembly Felis_catus_6.2^49^. The SNP positions are presented as IDs on the array and a map position for both cat genome assemblies is presented in Supplementary Data File 4. The array average marker distance is 37,741 bp, with a range of an average 36,699 bp between markers on chromosome D2 to an average 46,697 bp between markers on the chromosome X (Supplementary Table 1 and Supplementary Figure 1). The largest gap was detected on chromosome B2 (~3.2 Mb) followed by two markers on chromosome B1 (at ~3 Mb and ~2.5 Mb, respectively). The number of gaps >100 Kb is 1540 comprising 232 Mb, the number of gaps >500 Kb is 20 comprising ~26 Mb (Supplementary Data File 3).

### Animals

Table 1 presents the 47 breeds, populations and familial cat groups represented by the 2,078 DNA samples genotyped on the 63K cat array. The dataset included domestic cats (n = 1,570) from 41 breeds and two cross-breed pedigrees^49,51^, two lions (*Panthera leo*), two tigers (*Panthera tigris*), nine leopard cats (*Prionailurus bengalenesis*), and 60 European wildcats (*Felis silvestris ssp*). Three breeds were sampled from familial lineages for pedigree studies, including Birman^52^, Lykoi, and Tennessee Rex. The samples from a mixed-breed research colony of known matings were included to support segregation analyses of the SNPs^49^. Each sample had a genotyping rate ≥90%. Complete genotype data is found in Supplementary Data File 5. The eight cats used for SNP discovery were included in these analyses^46^. The multi-dimensional scaling (MDS) clustering (see below) suggested the sample provided and identified as a Burmese (Pixel) was switched with the sample identified as a Cornish Rex (Tipper)^47^.

**Table 1:** Population statistics and linkage disequilibrium (LD) estimates of cat breeds and populations.

Breed Name	Symbol	No.*	No. LD SNPs^†^	LD (kb)	% Mono	MAF	H_O_	F_IS_
Abyssinian	ABY	41*	36251	1050	26.6	0.16	0.20	0.06
American Curl	ACURL	25*	42704	200	17.5	0.19	0.25	0.03
American Shorthair	ASH	2	—	—	—	—	—	—
American Wirehair	WIR	9	—	—	35.4	0.16	0.25	−0.12
Asian	Asian	3	—	—	—	—	—	—
Bengal	BEN	98*	41053	350	11	0.18	0.23	0.05
Birman	BIR	296*	34068	1450	17.1	0.15	0.2	0.05
Bombay	BOM	11	—	—	20.5	0.2	0.25	0.07
British Shorthair	BSH	22	40503	250	19.9	0.18	0.24	0.02
Burmese	BUR	106*	32131	700	30.6	0.14	0.17	0.12
Chartreux	CHR	7	—	—	33.7	0.17	0.16	−0.09
Cornish Rex	CREX	11	—	—	27.5	0.17	0.23	0.03
Devon Rex	DREX	21	39562	500	19.6	0.18	0.22	0.09
Egyptian Mau	EGY	10	—	—	26.3	0.18	0.25	−0.04
Havana Brown	HAV	1	—	—	—	—	—	—
Japanese Bobtail	JBOB	13	—	—	20.2	0.2	0.27	−0.016
Khao Manee	MANEE	5	—	—	41.8	0.15	0.22	−0.08
Korat	KOR	6	—	—	55.5	0.11	0.17	−0.1
Kurillian Bobtail	KBOB	1	—	—	—	—	—	—
LaPerm	PERM	66*	45805	100	7.5	0.2	0.27	0.009
Lykoi	LYK	27	—	—	23.1	0.19	0.28	−0.12
Maine Coon	MCOON	54*	43748	150	12.3	0.19	0.25	0.025
Manx	MANX	8	—	—	20.4	0.2	0.28	−0.02
Munchkin	MUNCH	40*	47557	50	9.1	0.21	0.29	−0.007
Norwegian Forest cat	NFC	15	—	—	15.1	0.2	0.27	0.03
Ocicat	OCI	5	—	—	37.7	0.16	0.24	−0.1
Oriental	ORI	56*	35398	300	20.1	0.16	0.2	0.046
Persian	PER	153*	41893	150	11.4	0.18	0.23	0.07
Peterbald	PBALD	31*	38776	300	22.2	0.17	0.24	−0.05
Ragdoll	RAG	51*	42927	250	10.4	0.19	0.25	0.05
Russian Blue	RBLUE	6	—	—	32.8	0.17	0.22	0.04
Scottish Fold	SFOLD	150*	43182	150	8.1	0.2	0.25	0.05
Selkirk Rex	SREX	22	42131	150	17.6	0.19	0.25	0.016
Siamese	SIA	66*	33711	400	26.5	0.15	0.19	0.063
Siberian	SIR	51*	47587	50	7.1	0.21	0.28	0.007
Singapura	SIN	4	—	—	—	—	—	—
Somali	SOM	6	—	—	32.9	0.17	0.23	−0.005
Sphynx	SPH	26	42551	200	18	0.19	0.25	0.03
Tennessee Rex	TREX	21	—	—	—	—	—	—
Turkish Angora	ANG	4	—	—	—	—	—	—
Turkish Van	VAN	20	47820	50	11.7	0.2	0.27	0.026
**Total Breeds**	**41**	**1570**						
Domestic	DOM	262*	50544	<50	2.2	0.22	0.27	0.096
Wildcat	FSI	60	13059	750	36.2	0.05	0.06	0.24
Colony	Colony	139	—	—	10.9	0.2	0.27	0.0015
Oriental/Toygers	HYD	34	—	—	22.2	0.17	0.24	−0.07
Big Wild Cats	BIGW	4	—	—	—	—	—	—
Asian Leopard Cats	ALC	9	—	—	94	0.008	0.012	−0.05
**Total cats**	**47**	**2078**						

^*^Sample size reduced to 25 most unrelated cats within a breed or population except for the wildcats for LD estimates. No LD was estimated for populations less than 20 individuals or the pedigree populations (Colony, TREX, LYK, and HYD), including 21 populations represented by 597 individuals.

^†^SNP number does not include X chromosome SNPs.

The reported values for MAF, observed heterozygosity, and inbreeding coefficient are means.

### Genotyping accuracy, Mendelian errors and summary statistics

The comprehensive dataset of cats (n = 2,078) had a genotyping rate >90%. The array’s SNPs (n = 62,897) were evaluated for genotyping quality. Only ~1% (n = 625) of the SNPs were missing ≥10% of genotypes and were therefore excluded from downstream analysis (Supplementary Data File 6). The remaining SNPs were examined for Mendelian segregation using 86 trios from the research colony cross-breed pedigree^49^. All samples represented in the trios exhibited Mendelian errors in ≤2% of the markers, supporting familial relationships. Marker-specific Mendelian errors were identified for 232 SNPs, and each showed ≥10% errors (Supplementary Data File 7). Eleven of the SNPs with Mendelian errors also had a genotyping rate ≤90% across all samples, therefore, the total SNPs excluded were 846, leaving 62,051 SNPs for downstream analyses. The SNPs with Mendelian errors were assigned to the “unknown” chromosome (Chr 20) for a future potential use. Considering that X–linked SNPs could be located either in the pseudo-autosomal region where males could be heterozygous, X chromosome SNPs (n = 160) showed male heterozygote genotypes (errors) in ≥10% of 52 males within the trios (Supplementary Data File 8).

The feline array had an average SNP genotype call rate of ~99% in the 2,039 (98%) samples. Twenty cats were genotyped in replicas, including four samples replicated from the same DNA aliquot but genotyped on different arrays, one sample as a whole genome amplification, two samples represented by tumor tissue of the genotyped cat, and 13 samples replicated as part of separate studies from different DNA aliquots. SNP mismatches between repeated samples were calculated after removing SNPs with a genotyping rate ≤90% and after removing SNPs with Mendelian errors. The average mismatch between samples repeated from the same aliquot of DNA was 0.14%, ranging from 0 to 0.55%. However, the sample with the highest mismatches was a commonly used cat cell line (CCL-94; ATCC). The whole genome amplified DNA had 2.62% mismatches from the non-amplified DNA sample. The two samples represented by the tumor versus non-tumor tissue had 0.69% and 1.06% mismatches. The replicated samples from different DNA aliquots had an average of 0.48% mismatches, ranging from 0.07% to 0.85% (Supplementary Table 2).

After removing SNPs with a genotyping rate of ≤90%, all markers were evaluated for minor allele frequency (MAF) across all samples. None of the SNPs with low genotyping rates were of wildcat origin. Only 752 SNPs were monomorphic in all genotyped individuals, with the highest number of monomorphic SNPs on chromosome A1 (n = 89) and the lowest on chromosome E3 (n = 11) (Supplementary Data File 9). Additionally, 7,813 markers displayed a 0 < MAF ≤ 0.05, including, 2,628 markers with a 0 < MAF ≤ 0.01 (Supplementary Table 3). Overall, 59,423 SNPs (95%) on the cat array displayed high quality genotypes, proper Mendelian inheritance, and polymorphism across cat populations.

Four wild felids that were genotyped represent the most distant lineage from the domestic cat, including two lions and two tigers both from the *Pantherine* lineage^4–6^. These felids also had a per individual genotyping rate ≥90% and over ≥90% SNPs were successfully genotyped in the four wild felids combined. The *Pantherine* cats (BIGW, n = 4) exhibited very low polymorphism and only 1,754 SNPs were polymorphic. No genotypes were obtained from 3,733 SNPs for the large wild felids (BIGW). Asian Leopard cats (ALC, n = 9) were polymorphic for 3,547 SNPs. The European wildcats (n = 60) possessed a considerably higher number of polymorphic markers (n = 40,445). For the wildcat-specific SNPs, 2,576 of 4,240 (61%) were polymorphic with a MAF ≥ 0.05 within the domestic cats. In the *Pantherine*, 116 (2.7%) wildcat-specific SNPs were polymorphic.

### Cat population structure analyses

**Figure 1:**
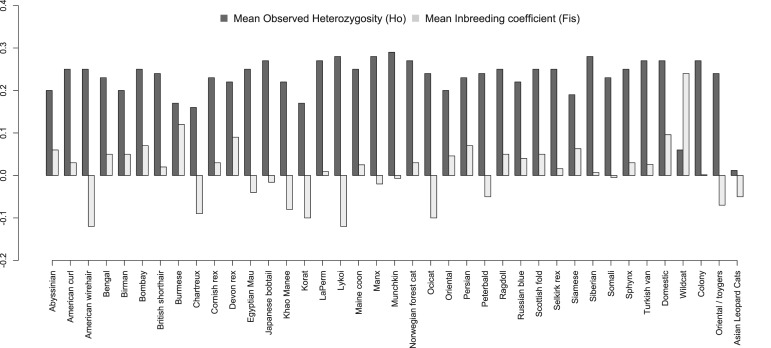
Summary of population genetics of cat breeds and populations. Random bred cats have the highest measures of genetic variation whereas several breeds have critically low genetic variation, such as Burmese and Birman. Breeds that have been developed more recently from random bred populations, such as Siberians and Munchkins, have high diversity, as well as breeds continually pulled from random bred populations, such as the Manx cats from the Isle of Man. Note: Domestic group represents random bred samples where as Oriental/Toyger is pedigree.

Breed-specific population summaries are presented in Table 1, Fig. 1. The average MAF across breeds and populations (excluding non-domestic cats) was 0.21. The LaPerm, Lykoi, Manx, Munchkin, and Siberian breeds had a slightly higher percentage of SNP heterozygosity compared to other breeds. Depending on cat breed, the percent of monomorphic SNPs were as low as 7% in the LaPerm cats (n = 4,659) and as high as 50% in Korat cats (n = 34,542). The mean MAF ranged from 0.11 in Korats and a high 0.22 for random bred cats, while the observed heterozygosity ranged from 0.16 for Burmese and Korats to 0.28 for Siberians. The population with the lowest number of monomorphic markers was the domestic shorthair population, which is believed to most closely mimic random bred cats, with only 1,410 non-informative markers (2%). The inbreeding coefficient (*F*_*IS*_) for the cat populations ranged from −0.12 for the Lykoi and American Wirehair breeds to 0.12 for the Burmese. Random bred domestic cats had an (*F*_*IS*_) of 0.096.

**Figure 2:**
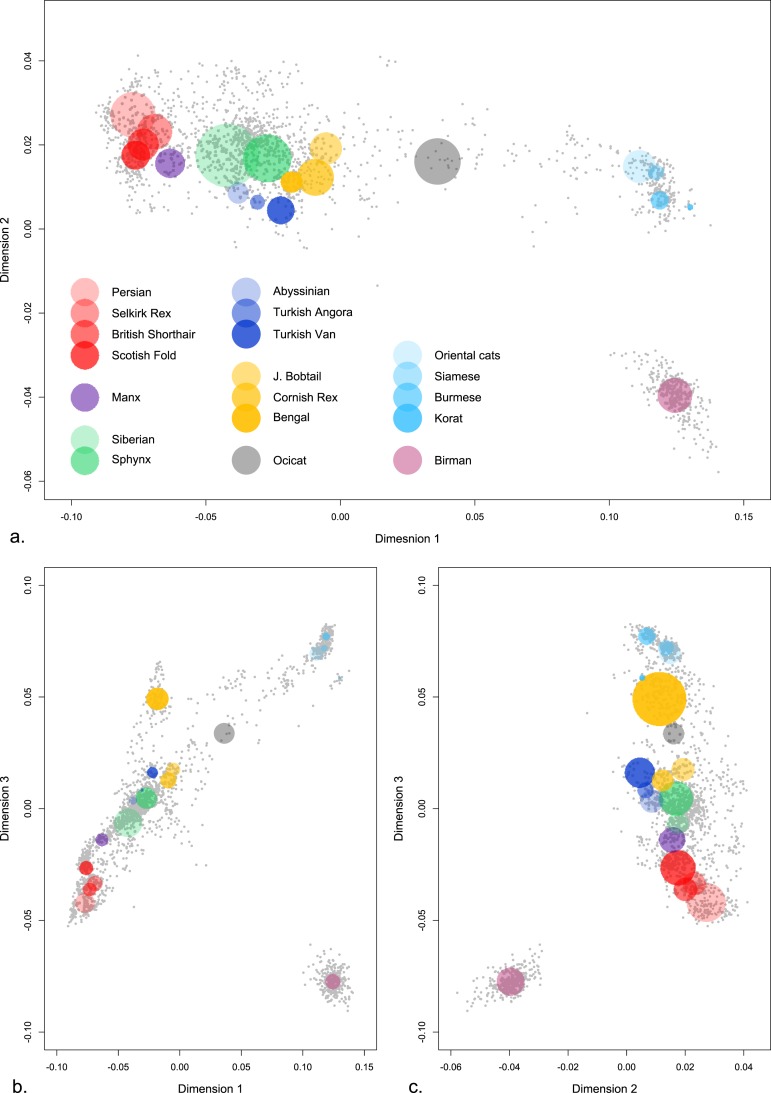
Multi-dimensional scaling of cat breed genetic structure. Plots of the genetic distances between individual domestic cats in three dimensions (C1 *vs*. C2, C2 *vs*. C3, C1 *vs*. C3). Gray dots represent individual cats and collectively show the overall distribution of populations. Selected breeds are highlighted by a colored circle where each colored circle corresponds to a population. The positions of the circles and the sizes are drawn to qualitatively distinguish between popular cat breeds (see materials and methods). (**a**) dimension 1, (**b**) dimension 2, (**c**) dimension 3. The Birman breed (light purple) consistently is a highly distinctive population. Asian breeds (light blues) are highly distinct from Western breeds (reds). Ocicat (grey) are a breed developed by crossing Abyssinians with Siamese and are intermediate in the gradation of cat breeds. The MDS of each population is presented in Supplementary Figure 4.

To visualize the relationship within and among cat breeds, all of 2,078 cats were assessed for population structure by multi-dimensional scaling (MDS). The MDS was performed using the 62,272 SNPs that had a call rate ≥90%. To illustrate breed structure, key breeds are highlighted in Fig. 2. Domestic cats were interspersed across all populations but a clear Western-Eastern distribution of the breeds was observed (Supplementary Figure 2a–c). Cat breeds with eastern origins, such as, Oriental, Siamese, Burmese, Korat and Birman, clustered at one side of the MDS plot, whereas, at the opposite extreme of the plot, the Persian breed family including, Persian, Selkirk Rex, British Shorthair, and Scottish Fold, clustered tightly at the opposite extreme of the plot. The Eastern-Western divide was observed on every combination of dimensions. The majority of the breeds clustered towards the Persian breeds (Fig. 2). Cats with Mediterranean origins, such as Turkish Angora, Turkish Van and potentially Abyssinians, formed groupings midway between the Eastern-Western origin breeds.

**Figure 3:**
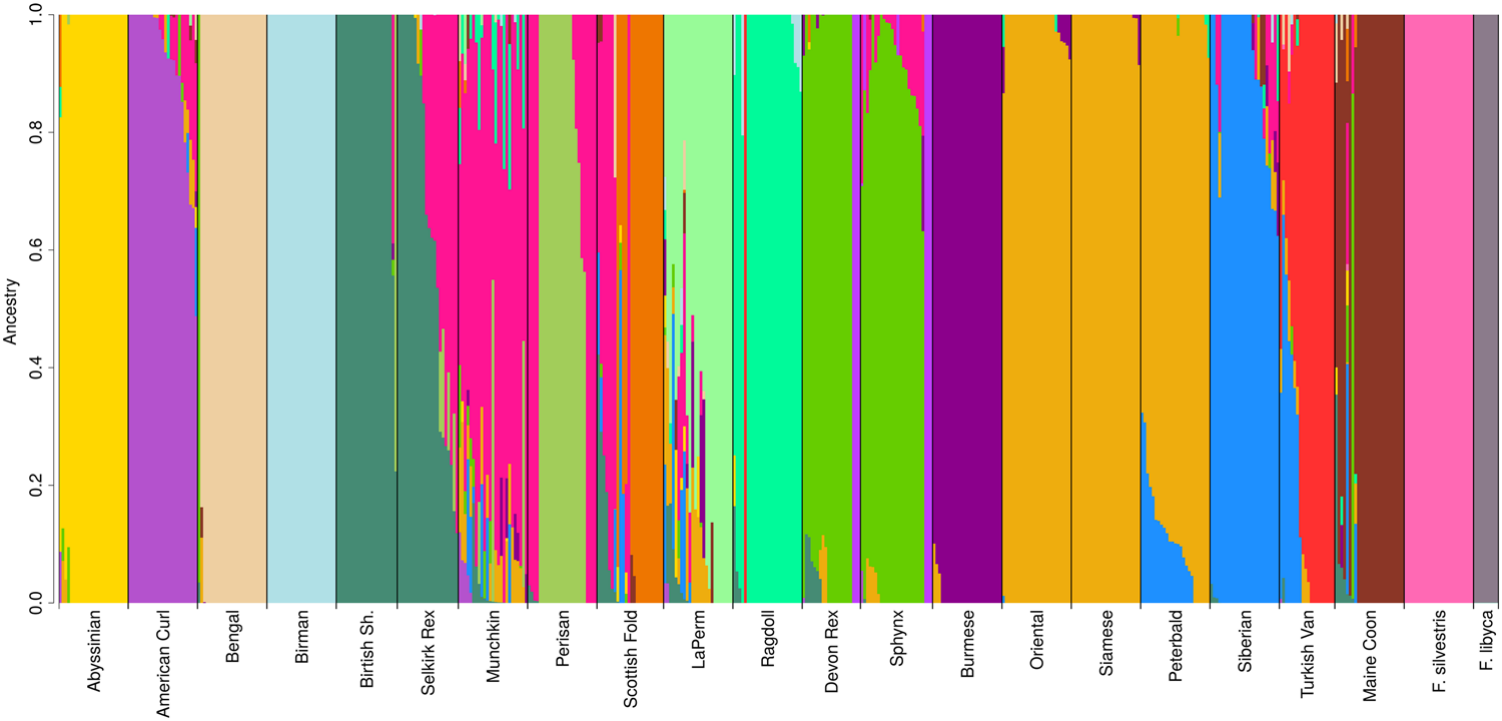
Population structure plot (K = 19) of twenty cat breeds and two wildcat populations. faststructure was used to examine the same cat populations as described for the MDS analyses. Cat breeds with the same colors indicate admixture and shared ancestry/cross-breeding. For examples, Peterbalds are derived from Siamese and Oriental lines and several breeds have been developed from Persian lineages, such as Munchkins and Scottish Fold, to obtain brachycephalic head structure.

Additionally, the cat breed population structure was investigated using the Bayesian fastSTRUCTURE^53^ analysis. Approximately 99.99% of the genetic variation (*K*_∅_*c* statistic in fastSTRUCTURE) among the twenty cat breeds and two wild cat (*F*. *silvestris* and *F*. *libyca*) was explained by a K = 19 (Fig. 3). The ancestry profiles of the cat breeds follow a similar pattern as the MDS (see above) where Eastern breeds such as Oriental, Siamese, and Peterbald shared over 60% of their ancestry assignment to a common cluster. Similarly, the closely related Western breeds, British Shorthair and Selkirk Rex, displayed a clear-shared ancestry, including sharing of Persian lineages that are also common to the Scottish Fold and the Munchkin breeds. Breeds that were developed within the past 30 years, such as LaPerm and Munchkin, showed higher levels of admixture when compared to older established breeds, such as, Birman and Burmese.

### Linkage disequilibrium

The genome-wide extent of linkage disequilibrium (LD) was measured using the squared correlation coefficient (*r*^2^) between pairs of autosomal SNPs on each chromosome, independently. Only SNPs with MAF ≥ 0.05 were included in the analysis for each breed, separately, therefore the number of markers varied between breeds (Table 1). Initially, the LD estimates were compared across five subpopulations of random bred cats (n = 10, 25, 50, 100 and 200 samples). The greatest difference in the *r*^2^ estimates was observed between a sample size of 10 and 25 (Supplementary Figure 3 and Supplementary Table 4). Therefore, further LD analyses and the Bayesian structure analyses were conducted on only populations with ~25 unrelated individuals.

**Figure 4:**
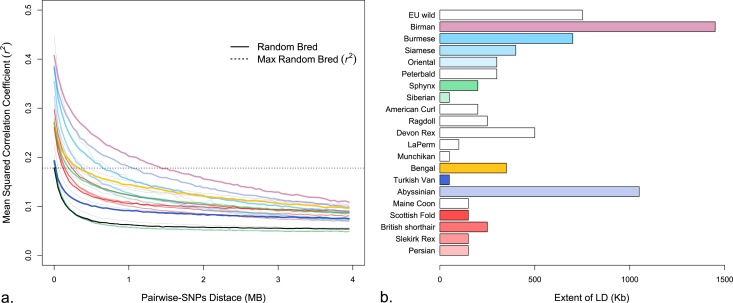
Genome-wide estimate of linkage disequilibrium (LD) of cat breeds. (**a**) Decay of LD (*r*^2^) at different bins of inter-SNP distances. LD decay of selected population is shown as a color (see (**b**) for key to colors) and remaining populations are shown in gray. Solid black decay line corresponds to the random bred population, to which all breed populations are compared. Horizontal dotted line represents the maximum of *r*^2^ value in random bred population and the point of comparison between populations (the point of LD < 50 Kb). (**b**) Extent of LD (Kb) where the *r*^2^ value reaches that of random bred population.

The genome-wide LD was estimated for twenty cat breeds, random bred cats and the European wildcat population (Fig. 4a and Table 1). As a measure of the extent of LD and to allow cross-population comparison, the maximum *r*^2^ value for the domestic cat (DOM) population was used as the cutoff point and the *r*^2^ value of comparison. Genome-wide LD among cat breeds ranged from 50 Kb in Munchkin, Siberian and Turkish van to a maximum of ~1,500 Kb in Birman cats. (Table 1, Fig. 4b and Supplementary Table 4). In general, Eastern breeds, which include Birman, Burmese, and Siamese, exhibited a larger extent of LD (1450, 700 and 400 Kb, respectively). The Persian family of breeds, which includes Persian, Selkirk Rex, British Shorthair and Scottish Fold, showed an intermediate extent of LD 150–250 Kb with little variation among the breeds. The Siberian, Munchkin, and Turkish van breeds displayed the lowest levels of LD at 50 Kb. The European wildcat population displayed an LD of 750 Kb.

### Genome-wide association analyses

To evaluate the power of the feline array for localizing traits via association analyses in cats, four aesthetic traits were chosen based on sufficient phenotypic documentation in the dataset. Of the four traits, three are inherited in an autosomal recessive fashion, specifically coat color loci *Dense*^54^, *Color*^55–57^, and the fur type *Long*^58,59^, and the X-linked *Orange* coloration locus^43,60^. Causative variants of the three autosomal traits were previously identified and were included on the array. The causative variant of X-linked *Orange* color is still unknown. The presence of the three phenotypic SNPs on the array allowed measuring the power of association under different population conditions (size or heterogeneity), in the presence or absence of artificial selection and allowed a comparison of association of the causative variant and adjacent SNPs. The SNPs associated with each trait and *P*_*genome*_ values after permutation testing are presented in Table 2. The SNPs with the highest association to the traits are presented in Supplementary Table 5. All association studies remained genome-wide significant after permutation testing. Genomic inflation values are reported in Table 2.

**Table 2:** Genome – wide associations to determine power of the cat DNA array.

GWAS	MOI	Cases	Controls	ʎ	Chr.	Position	Haplotype length	*P*_*raw*_	*P*_*genome*_	SNPs post mperm
*Dense* ^*,†^	AR	33	81	1.06	C1	218,100,114	NA	1.30e^−20^	0.00001	1
- Burmese	AR	30	56	1.46	C1	218,200,114	~150 Kb	3.79e^−16^	0.00002	15
- Birman	AR	60	41	1.24	C1	218,100,114	~60 Kb	8.08e^−20^	0.00002	13
Long hair*	AR	32	22	1.17	B1	140,077,554	~150 Kb	8.20e^−10^	0.00010	2
*Color* (*c*^*s*^)*	AR	21	28	1.41	D1	46,341,460	~1 Mb	2.00e^−9^	0.00040	10
*Orange*	X	24	69	1.11	X	107,777,134	~1.5 Mb	1.20e^−20^	0.00002	7

^*^Causative variant is present on the array. ^†^The power to detect *Dense* was first considered for random bred cats and then for breeds in which the trait is under selection.

#### Autosomal recessive trait in the random bred population

**Figure 5:**
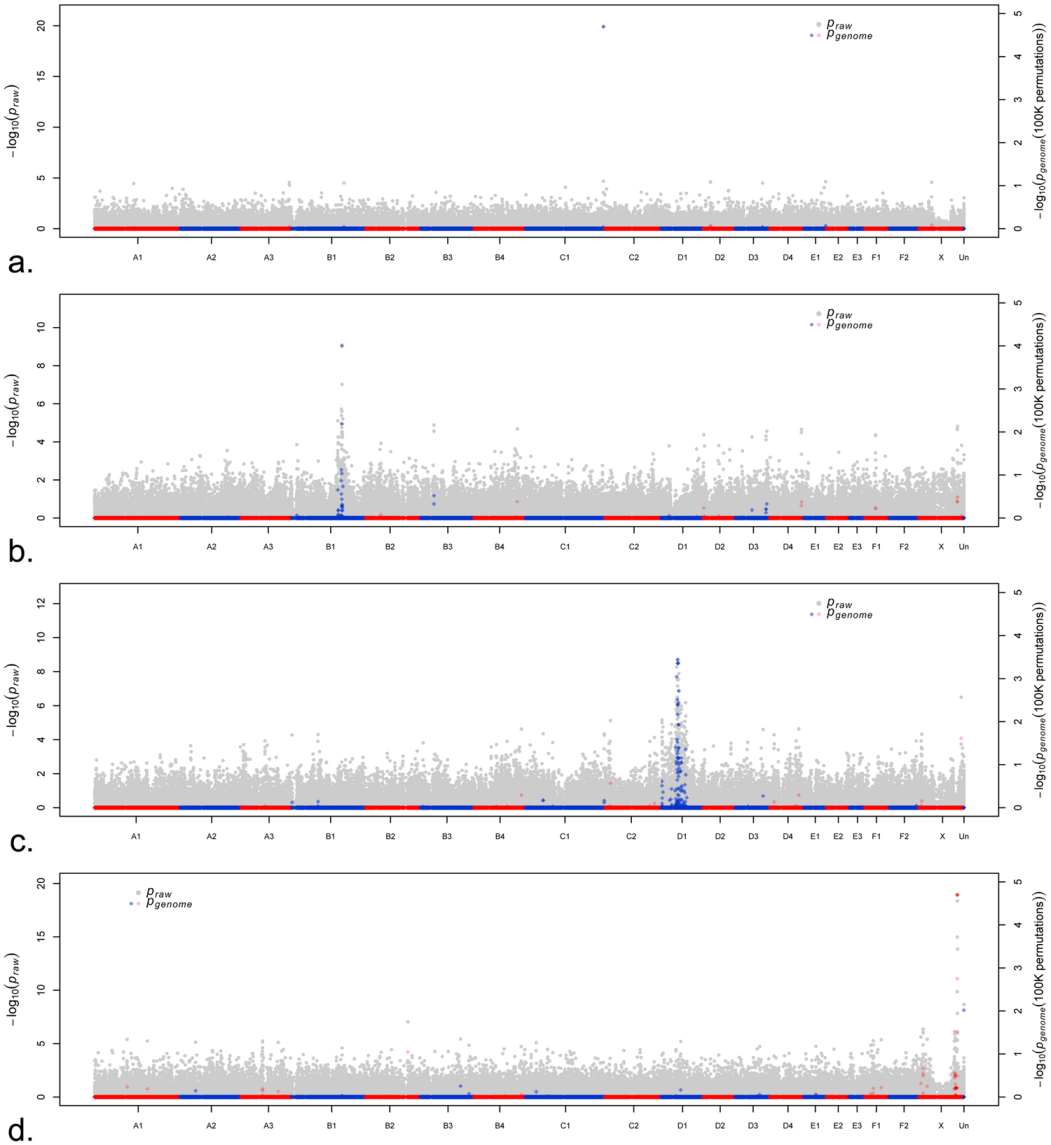
Illustrative genome-wide association analyses for four phenotypic traits in the domestic cats. Manhattan plots of the association analyses where x-axis represents chromosomes, gray dots and left y-axis represent raw P-values of the association, and red/blue dots and right y-axis represent the permuted P-values. (**a**–**c**) Remapping of three autosomal recessive traits (*Dense*, *Long*, and *Color* (*c*^*s*^ allele), respectively) and (**d**) X – linked *Orange* using different populations. (**a**) Only the causal SNP for *Dilute* is associated in random bred cats on cat chromosome C1. (**b**) Several SNPs are associated with the long hair phenotype on chromosome B1 in LaPerm, a newer breed but with little selection for the trait. (**c**) Several SNPs are associated for the *c*^*s*^ allele in *Color* on chromosome D1 in Persians, one of the oldest breeds where the coloration has some positive selection. (**d**) GWAS of *Orange*, an X-linked trait, suggesting a critical region for the locus.

Thirty-three cases and 81 controls of domestic cats were selected for the association of *Dense* (*a*.*k*.*a Dilute* coat color), a trait not under selection in random bred cats (Table 2 and Supplementary Table 5). A single significant SNP, located on chromosome C1 at position 218,100,114, was associated with the phenotype (raw *P*_*value*_ = 1.3e^−20^), which is the causal variant within *Melanophilin* (*MLPH*)^54^ (Fig. 5a). For the closest SNP to the *MLPH* causative variant to show a significant association, the number of samples would need to be increased from 114 to 427 when using the current density array (Supplementary Figure 4a). The flanking SNPs were 39 and 22 Kb from the causal SNP.

In comparison to the *Dense* association in random bred cats without selection, 30 cases and 56 controls were used to perform the same GWAS within the Burmese breed and 60 cases and 41 controls within the Birman breed with selection for the trait. Several SNPs detected association together with the causal variant (raw *P*_*value*_ = 3.79e^−16^ in Burmese and raw *P*_*value*_ = 8.08e^−20^ in Birman). While the analysis with random bred samples showed an association only with the causative variant, Burmese exhibited a ~150 Kb haplotype block (position 218,100,114–218,250,626) and Birman had a ~60 Kb haplotype block (position 218,060,712–218,122,590) across all cases. This comparison showed how an association analysis within a breed with positive selection for a trait is likely to be more successful than in a random bred population. Furthermore, while achieving an association of the closest marker with the causative variant in random bred cats requires significantly increasing the number of samples or markers, an association can be detected in Burmese even when reducing the number of samples from 101 to 37 (13 cases and 24 controls); the most significantly associated SNP remained statistically significant (*P*_*value*_ = 1.05e^−6^) after permutations (*P*_*genome*_ 0.009). In Burmese, a unique haplotype of ~4.5 Mb containing the causal variant for the phenotype was detected across all cases while in Birman a unique haplotype of ~200 Kb containing the causal variant was identified in all cases. The SNP composition of the Birman and Burmese haplotypes were different, including within the 200 Kb haplotype that is within the 4.5 Mb haplotype of the Burmese.

The Chartreux, Russian blue, and Korat breeds are fixed for the variant in *Dense* and the region of homozygosity for these cats extended 190 Kb in Chartreux and Russian blue and 280 Kb in Korat.

#### Autosomal recessive trait in a breed, without selection

The LaPerm breed is characterized by its curly coat texture and comes in both longhair and shorthair varieties^20^. However, only the curly coat texture is consistently selected in the breed while the longhair variant is not under selection. LaPerm breed displayed low LD (100 Kb) and high polymorphism (7.5% monomorphic SNPs). Thirty-two cases (longhair) and 22 controls (shorthair) of the LaPerm breed were selected to perform a GWAS for the longhair trait. (Table 2, Supplementary Table 5). The most common causative variant for longhair is in *fibroblast growth factor* 5 (*FGF*5)^58,59^, which is located on chromosome B1 (at position 140,077,554 of the 6.2 genome assembly)^46^. The *FGF5* causative variant was the most significantly associated with the hair length phenotype (raw *P*_*value*_ of 8.2e^−10^), in addition to several other adjacent SNPs (Fig. 5b and Supplementary Figure 4b). For the closest SNP to the causative variant within *FGF5* to have similar association power, the number of samples would need to be marginally increased from 54 to 66 cats.

Breeds fixed for longhair include Maine Coon, Norwegian Forest Cat, Persian, Ragdoll, Siberian and Turkish Angora. The regions of homozygosity surrounding *FGF5* in these breeds flanked the causal variant for longhair on the array by 382 Kb, while the length of the haplotype block in the LaPerm breed was 150 Kb.

#### Autosomal recessive trait in a breed and under selection

Pointed cats have a variant at the *Color* (*c*) locus within *Tyrosinase* (*TYR*) and have a darker coat color on the ears, face, paws and tail^55^. Using pointed (*c*^*s*^*c*^*s*^) Persian cats (a.k.a. Himalayans) as cases and non-pointed Persian cats as controls (Table 2 and Supplementary Table 5), many significantly associated SNPs were identified on chromosome D1 near position 46,341,460 (Fig. 5c and Supplementary Figure 4c). The power of SNPs in the *TYR* region to detect association was very similar to that of the causative variant due to complete linkage between markers. To obtain the same power as the causative variant using adjacent linked SNPs, the number of samples would need to be increased from 49 to 50. The length of the haplotype block containing the variant in the Himalayan cases was 1 Mb. Points are fixed for *c*^*s*^ allele in Siamese and Birman, and the *c*^*b*^ allele in Burmese and the haplotype block is 430 Kb, 480 Kb and 4.2 Mb, respectively.

#### X-linked trait in a cross - breed analysis

The X-linked *Orange* coloration^43^ was localized using cases (24) and controls (69) from multiple breeds from the dataset as previously described in Gandolfi *et al*.^61^. *Orange* was localized to the X chromosome by allelic association (most significantly associated SNPs at positions 107,777,134 and 107,994,240 with raw *P*_*value*_ = 1.8e^−19^ and 4.3e^−19^, respectively), and by Cochran-Mantel-Haenszel test (CMH) to position 107,777,134 with raw *P*_*value*_ = 4.4e^−5^ (Table 2, Fig. 5, Supplementary Table 5 and Supplementary Figure 5). The associated markers reside in the same linkage region identified previously^43^. A haplotype for *Orange* was evaluated by exporting 5 Mb of genotypes, from position 105 Mb to position 109 Mb of the X chromosome (107 SNPs). A haplotype block was detected from position 106,241,242 to position 107,745,900 (~1.5 Mb) of the X chromosome (Supplementary Figure 6). The haplotype block contains 12 genes, listed in Supplementary Table 6.

## Discussion

Low-density genotyping arrays are available for a variety of species. The design of the feline array benefitted from the results and outcomes from the designs for dog^62^, cow^63^, pig^64^ and horse^65^. At the time of SNP selection the cat genome assembly was not as robust as these other species, however, the selection of widely diverse cat breeds and domestic cats from diverse regions of the world supported the identification of >10 million SNPs for array design^47^. The final array contains ~63K variants, the highest number of SNPs when compared to the first-generation equine (54.6K), canine (49.6K) and bovine (58.3K) arrays^63,65,66^. This low density array is highly suitable for Mendelian trait analyses, particularly in cat breeds.

The position of the SNPs was based on the feline genome assembly FelCat 4 (*Felis catus 5*.8). After SNP remapping to the latest feline genome assembly FelCat 8.0 (*Felis catus* 8), only 704 SNPs (1.1%) remained unassigned, a significant improvement from remapping to cat Felis_Catus_6.2 by Alhaddad *et al*.^49^, where 6,893 SNPs had unknown locations. Marker coverage on the X chromosome is not as robust, likely due to the complexity of the X chromosome and the high density of repetitive sequences^67^. The feline inter-marker average distance of 37.7 Kb is equivalent to cattle^63^ and denser than the horse array, which has a ~43 Kb inter-marker distance^65^. The cat, cow, and horse genomes (2.64 Gb, 2.70 Gb, 2.42 Gb, respectively) are roughly equivalent in size. Although the feline genome assembly contains several gaps (~40 Mb) and unplaced scaffolds^46^, the inter-marker distances suggest balanced and slightly better coverage of the cat genome than for other species with early lower density arrays. However, the 20 gaps >500 Kb in the cat SNPs is higher than horse, with only 12 gaps >500 Kb and cow, where the highest gap between SNPs is <350 Kb^63,65^.

The cat array demonstrates a very low number of SNPs with low genotyping rate (625 SNPs, <0.01%) across ~2,000 samples, a low number of SNPs with Mendelian errors (n = 232, 0.004%), leaving 62,051 robust SNPs for downstream analysis. The number of SNPs excluded for low genotyping rate and Mendelian transmission errors is lower than that of cow and horse (0.09% and 0.05%, respectively)^63,65^. Thus, exclusion of ~1K SNPs for the array analysis is comparable to other first-generation arrays^63,65^.

Moreover, the presence of duplicate controls confirms the high reproducibility of the genotypes, with a negligible number of errors between replicates from the same aliquot of DNA. Slightly higher mismatch rates were observed in tumor versus genomic DNA and a cell line, both likely due to somatic mutation heterogeneity. The error rate between WGA samples and the original sample was 2.62%. Thus, excluding SNPs from analyses with a MAF ≤ 0.03 instead of the typical 0.05 may be acceptable. The removal of poor quality SNPs did not significantly affect mismatch rates. The mismatch rate is 10-fold higher than reported in cattle^63^.

The average MAF was variable across breeds, ranging from 0.11 for Korats to 0.22 for random bred cats. The average MAF of domestic cat populations was 0.18, which is lower than cows (0.26)^63^ and horses (0.24)^65^. Specifically, 2,628 SNPs (~4%) showed a MAF < 0.01 across all samples. This observed MAF is lower than other species, and is likely due to inclusion of SNPs that were specific to one wildcat species.

Although a Burmese cat was used as part of SNP sequencing discovery panel^47^, the percentage of monomorphic SNPs was the highest, at ~31%. For Burmese, the low number of polymorphic SNPs confirms the high inbreeding coefficient in the breed and inbreeding history^68,69^. A high number of monomorphic SNPs were observed in the large wild felids of the genus *Panthera* (lions and tigers; 94%), which is consistent with previous reports^8^. Even with limited numbers of polymorphic SNPs on the array for large wild felids, the remainder of polymorphic SNPs can be used for conservation and zoo management applications. A substantial number of SNPs (63.8%) are informative for European wildcats. These thousands of polymorphic markers may be useful for population and conservation studies, especially in wildcat subspecies^70^. However, the cat 63K array is unlikely to be useful for disease mapping studies in distant wild felids.

The MDS clustering and Structure analyses confirmed the known origins of the cat breeds and their relationships^68,69^. The cat breeds displayed a continuum on the MDS plots, however, three main clusters are observed representing cat breeds with Western, Central and Eastern origins. The Western breeds were represented mainly by the Persian family^71^, clustering in the second and third dimension as well, confirming a strong Persian genetic influence in British shorthair, Selkirk Rex and Scottish Fold, and in agreement with previous STR and SNP based studies^71,72^. Previously unstudied breeds, such as American Curls and Peterbalds demonstrated their Western and Eastern origins, respectively.

Breeds with Eastern origins (Birman, Havana Brown, Khao Manee, Korat, Oriental Shorthair, Peterbald, Siamese and Singapura) are found at the opposite end of the MDS and showed shared ancestry. The Birman cats are strongly clustered but genetically distinct from other Eastern breeds. The difference between the Birman clustering compared to results from previous study^61^ may be explained by the presence of a high number of related individuals that belong to mainly two big pedigrees of Birman cats. The Abyssinian breed clustered with the central origin breeds in the MDS that includes only domestic cats, specifically with Siberian in the 2^nd^ and 3^rd^ dimension. However, the close clustering with Siberian cats does not reflect the historical development of the breed. In previous studies, the Siberian breed was suggested to be genetically distinct from the other breeds^61,68^. The cross-bred Ocicat, an Abyssinian and Siamese hybrid, clustered in between the central and Asian breeds, showing both the European and/or Asian genetic influences^69,73^.

The present study represents the genome-wide LD estimation in cats and is in overall agreement with the previously reported estimates using selected regions^73^. The greatest difference of LD estimates (*r*^2^ values) was found between 10 and 25 samples of random bred individuals. As a result, LD was calculated for breeds and populations represented by at least 20 individuals. Eight breeds (Abyssinian, Birman, Burmese, Maine Coon, Persian, Siamese, Siberian and Turkish Van) and random bred cats displayed LD estimates that were similar to previously published results^73^. In contrast, a substantial difference in LD is evident for Abyssinian and Birman cats, where the LD was 10 and 7-fold higher, respectively, using genome-wide data. A significant difference was also observed in Siamese, where the LD was estimated at almost twice as long (400 Kb *vs* 230 Kb) as detected in the previous study. The discrepancy in LD estimates for these breeds is likely related to the size of region and number of SNPs used. Overall, Eastern breeds tended to have higher levels of LD (Birman, Burmese, Oriental shorthair, Peterbald and Siamese) relative to central and Western breeds.

The short LD of some cat breeds can be explained by (1) a large breeding population, such as Persian and Persian-derived breeds, (2) limited selection, whereby several possible coat colors are permitted (American Curl, LaPerm and Maine Coon), and (3) active outbreeding strategy (Munchkin), or random bred based breeds (Siberian). Persian and Persian-derived cats showed very similar levels of LD, as well as in Eastern breeds, such the Oriental Shorthair, which was used in the development of the Peterbald. The random bred population showed very low levels of LD, and breeds such as Munchkin, Siberian and Turkish Van displayed a haplotype structure similar to the random bred population, which is consistent with their breed history. Haplotypes length and LD levels also reflect the number of successful GWAS conducted in several cat breeds^49,72,74,75^.

The main application of a high-density array is the localization of simple Mendelian diseases and traits of interest. Using the presence of phenotypic SNPs on the feline array, several association scenarios were conducted and the power of the array was examined by comparing the p-values and LD of genotyped phenotypic SNPs (causative) to that of the surrounding SNPs. The first scenario was a GWAS for the recessive *Dense*^54^ trait that is not under selection using 114 random bred cats. As expected, the association identified only the causative variant (c.83delT in *Melanophilin* (*MLPH*)), and association analyses using random bred samples will require a denser array or a larger number of samples. When the same trait was analyzed using two breeds (Burmese and Birman) where the trait is under selection only in certain lines, a large haplotype block was associated using substantially fewer samples (n = 37) compared with random bred cats.

The second scenario using LaPerm cats identified a significant association of the most common *FGF5* variant (c.475A > C) for *Long* fur length^58^. The LaPerm breed is defined by and selected for curly coat texture but exists in longhair and shorthair varieties^20^. Despite the absence of positive selection for the variant, along with low LD, and high polymorphism within the breed, a significant association was detected with SNPs linked to the *FGF5* variant. Clearly, GWAS using cat breeds with traits under selection is more efficient than studies within random bred cats.

The third scenario analyzed the association of the *Color* mutation c.940G > A within *Tyrosinase* (*TYR*)^55,56^. The *TYR* variant is under positive selection in Himalayan cats, which have low LD and low inbreeding. A significant association was detected by multiple SNPs linked to the genotyped *TYR* variant and a haplotype block is shared among Himalayan cats.

The fourth scenario localized and refined the region of the unknown X-linked *Orange* locus^43,60^. The association analysis across breeds refined the region of association to a 1.5 Mb haplotype block. The region contains twelve genes, and after visual inspection of the genes and their function, a candidate was not apparent. Additional mapping efforts are required to refine the position of the locus and to identify candidate causal variant(s). This analysis, in addition to its contribution to refining the region of *Orange*, illustrates the efficiency of performing association analysis of X-linked traits, in random bred cats with no selection for the trait.

### Array success and applications

Preliminary predictions of the strength of population structuring and high LD in dog breeds suggested only 5,000 to 30,000 SNP markers were required to achieve complete coverage of the dog genome^76^, compared to an estimated 200,000 to 500,000 SNP markers in humans^77^, making GWAS in dogs both cheaper and easier to conduct^77,78^. Considering both the Illumina Canine SNP_20_ and the Affymetrix Canine V 2.0 Platinum Panel array, many GWAS in canines have been conducted with ~30 cases and controls. More complex traits^79,80^ obviously require more samples and hence the development of higher density arrays. Transmission distortion testing (TDT) has been successful with only 7–13 discordant sib-pairs in canine studies^81,82^. The feline array has also proven its utility within breeds and supported the genetic dissection of simple^49,61,72,75,83,84^ and complex traits^52,85,86^. The array clearly shows significant association power for traits under selection or recessive traits. Examples of successful GWAS for diseases include the frontonasal dysplasia in Burmese^84^, congenital myasthenic syndrome in Devon Rex^83^, and hypokalemia in Burmese^75^. Identifying the curly hair variant of Selkirk Rex and the variant for folded ears in Scottish Fold are examples of dominant traits that are under positive selection^72,74^. A comparable number of cases and controls have been used in these cat studies with minimal cases required for studies in the breeds with the highest LD, such as the Burmese. Many cat breeds are younger in breed development, such as Siberians, or still represent indigenous populations, such as the Manx cats on the Isle of Man, hence an association study in breeds with low LD more likely requires a higher number of samples or a denser array to provide a statistically significant association while analyses of random bred populations likely requires a significantly denser array.

Beyond the successful GWAS approaches presented here and published before, the feline SNP array enabled (1) the development of a high density linkage map^48^ that has supported the newer genome assembly, (2) an understanding of genetic variation within and between cat breeds^61,72^, (3) high resolution descriptions of genomic consequences of the selective sweeps^61,84^, and (4) a more fully refined comparative model for human biomedical research^83,84^.

## Materials and Methods

### Data availability

All data generated in the project is available in Supplementary information files included in the article for download.

### Ethical statements

Sampling of cats for this study was approved by the Animal Care and Use Committee (ACUC) of the University of California, Davis (protocol # 16991) and the University of Missouri (Protocol # 7808) and samples were collected in accordance with the guidelines and regulations. Samples were acquired by specialists in the field, such as veterinarians, or voluntarily donated by owners and breeders.

### SNP selection for array design

SNPs were identified from one cat of each breed representing American Shorthair, Cornish Rex, European Burmese, Persian, Ragdoll and Siamese, as well as one South African wildcat (*Felis silvestris cafra*)^47^. The re-sequencing efforts identified over three million polymorphisms with 964K common SNPs suitable for the design of a domestic cat genotyping array and 849K SNPs were likely to have an informative minor allele frequency >5% across cat breeds. Additional SNPs were identified from four pooled individuals representing six breeds, including Birman, Egyptian Mau (n = 1), Japanese Bobtail, Maine Coon (n = 5), Norwegian Forest cat and Turkish Van. Random bred cats with Eastern and Western origins, as well as two *Felis silvestris* and two *Felis libyca*, also assisted SNP identification^47^. Over nine million SNPs were identified from the deep re-sequencing of the cat genome.

A preliminary build of the cat genome, (FelCat 4, Felis Catus 5.8), was used to estimate spacing between SNPs. After exclusion of SNPs based on minor allele frequency (<0.25), near or within a sequence repeat, within a duplicated region, or with more than two alleles, approximately 1 million SNPs were submitted to Illumina for design of the DNA array. A vast majority of the SNPs have a one bead assay design and were mainly targeted as single copy, intergenic and intronic SNPs.

### Remapping array SNPs to the newest 8.0 cat genome assembly

To determine the exact coordinate of each variant in *Felis_catus*_8.0, the following analyses were performed. For each SNP, 100 bp of upstream and downstream sequence was aligned to *Felis_catus*_8.0 using the program blat^87^. The entire *Felis_catus*_8.0 reference sequence was used in the alignment rather than performing multiple alignments with separate chromosome sequences. The program was run in default mode to generate alignments, with a minimum of 11 bp of matching sequence to initiate an alignment (*tileSize* = 11) and at least 90% matching bases required (*minIdentity* = 90). The number of tile matches was 2 (*minMatch* = 2), the minimum score was 30 (*minScore* = 30), and the size of the maximum gap between tiles in a clump was 2 (*maxGap* = 2). The best matches were selected to determine the location of each pair of sequences (e.g., [upstream/downstream]) in the assembly and coordinates obtained. The remapped map file is available in Supplementary Data 2, which contains original SNP position and array identification number, the *Felis_catus*_6.2 position and the *Felis_catus*_8.0 position.

### Animals

A dataset comprised of 2,078 samples from 47 different groups/populations were genotyped on the Illumina Infinium iSelect cat array (Illumina, San Diego) as previously described^75^. The individuals from most populations were selected with minimal relationships $(\hat{{\rm{P}}} < 0.25)$ based on pedigree analysis for case-control analysis or population studies (Supplementary Figure 5). The Birman^52^, Lykoi, and Tennessee Rex breeds, as well as the Oriental/Toyger pedigree and colony cross-breed groups^51^, contained related individuals. The research colony cats were used for the segregation analyses^49^. PLINK^88^ was used to obtain the genotyping rate for each sample. Coat color, texture and fur length information were available for the majority of the samples genotyped.

### Genotyping accuracy, Mendelian errors and summary statistics

Quality control analyses for SNPs data were conducted using PLINK^88^. A dataset comprised of 2,078 samples were genotyped on the Illumina Infinium iSelect SNP array. SNPs with genotyping rate >90% across the dataset were identified using the command*–geno* 0.1.

A multi-generational cross-bred pedigree comprised of 86 trios (100 individuals – 52 males and 48 females) was used to determine marker-specific significant Mendelian errors^49^. Using the function*–mendel*, percent Mendelian errors per individual sample and per SNP were estimated. SNPs exhibiting ≥10% Mendelian errors were reported as significant errors. The distribution of SNPs with errors was investigated for each chromosome. Male-specific Mendelian errors or SNPs located in the pseudo-autosomal region of the X chromosome were determined by examining heterozygous X-chromosome genotypes in males (n = 52). SNPs exhibiting 10% or more of an X-chromosome in the males were reported as likely pseudo-autosomal SNPs.

Genotypic differences between replicates were analyzed for 20 samples. The genotypes of the original and the replicate samples were determined to be identical using the function (*identical*) in R base. The number of instances where a mismatch was detected were counted and presented. The number of discordant genotypes for each duplicated sample was determined across all SNPs (n = 62,897), after removing SNPs missing 10% genotypes (n = 62,272), and after removing SNPs missing 10% genotypes and with Mendelian errors (n = 62,051).

For each population independently, the following summary statistics were calculated using PLINK^88^, the function*–freq* was used to calculate (1) the number of monomorphic SNPs, and (2) the mean and standard deviation of minor allele frequency (MAF). (3) The mean and standard deviation of observed were obtained using the function*–hardy*. The number and frequency of all polymorphic SNPs (n = 62,272) for a dataset containing all domestic cat breeds combined was determined using the PLINK function (*–freq*). The numbers of SNPs within different minor allele frequencies bins are reported.

### Population inbreeding and structure analysis

The observed heterozygosity and the inbreeding coefficient were both calculated per individual using*–het* command in PLINK (v1.9) and the mean of the values for each population were reported.

To depict the genetic relationships between populations and individuals within each population, pairwise genetic distances between all individuals in the dataset were calculated in Plink^88^ using the*–genome* function. The genetic distances obtained were used to generate a multi-dimensional scaling of the genetic distances between individuals (using the command*–mds*-*plot*). Three dimensions were used to visualize the genetic population structure of breeds. Each population was plotted in relation to all other populations in three combinations of dimensions (C1 *vs* C2, C1 *vs* C3 and C2 *vs* C3). The entire dataset was plotted and open circles were used to show the position of the populations. The circles represent a qualitative depiction of the position of a population and drawn as follows. For each population (A), the position of the circle was determined by mean (dimension1), mean (dimension2), whereas the radius of the circle was chosen using the largest of the standard deviations of (dimension 1 or 2). Each of the three combinations of dimensions (C1 *vs* C2, C1 *vs* C3, C2 *vs* C3) was plotted separately.

Additionally, the utility of the array data in identifying levels of population admixture was examined via fastSTRUCTURE^53^ (version 1.0). To reduce the effects of uneven sample sizes between populations^89^, only unrelated samples from twenty breed and two wildcat populations (n = 519), which are equal in size (see populations used for LD analysis below) were used in the analysis. The autosomal SNPs of all samples were used and SNPs with a MAF less than 0.01 (n = 1198) were removed, which resulted in 57,690 SNPs to be used in the analysis. fastSTRUCTURE^53^ was run to determine the genomic contribution of K (K = 1–20) hypothetical populations. Two outputted metrics were considered to determine the appropriate values of K: (1) the K that maximizes the log-marginal likelihood lower bound and (2) the minimum value of K that accounts for 99.99% cumulative ancestry.

### Selection of unrelated samples and linkage disequilibrium analysis

To unbiasedly measure the genome-wide extent of linkage disequilibrium (LD) in cat breeds, a number of criteria were considered including, (1) the LD statistic, (2) number of individuals per breed, (3) degree of relatedness among individuals within a breed, and (4) the statistical point (*r*^2^ value) of comparison between breeds. The pairwise squared correlation coefficient (*r*^2^) was used as a measure of LD between any two autosomal markers on the same chromosome as previously described^73^. To assess the effects of sample size on the measure of the extent of LD, a dataset of domestic random-bred (DOM) cats (n = 270) was examined by randomly selected (without replacement) individuals to represent five populations of different sample sizes (specifically, 10, 25, 50, 100, and 200 individuals). For each of the DOM subgroups, *r*^2^ were calculated, as described above. The effect of the sample size was measured by comparing the *r*^2^ values between the five subgroups.

As an outcome of the assessment of the effects of sample size on LD measure (see results), only the breeds represented by 20–30 unrelated individuals were used in the LD analysis. To ensure unbiased measure of LD due to relatedness, the individuals representing each breed were selected based on the lowest identity by descent (IBD) values. IBD values were obtained using the command*–genome* using PLINK^88^. For each population independently, *r*^2^ was calculated for autosomal markers that exhibited (MAF ≥ 0.05) and analyses were performed using Haploview^90^.

Pairwise *r*^2^ estimates between autosomal markers on the same chromosome were jointly categorized into distance bins of 50 Kb. The range of distances between markers included in the estimation of the extent of LD was 50 Kb–4 Mb. In each distance bin, the mean LD estimate was used as the representative of the statistic. The decay of LD was determined by connecting the statistic *r*^2^ mean at every distance bin. To objectively report the extent of LD, the maximum value of *r*^2^ found in the random bred population was used as the *r*^2^ value of comparison. This *r*^2^ value represented lack of LD or extent of LD smaller than 50 Kb that is seen in the random bred population (DOM).

### Remapping of known coat colors loci using GWAS

Three autosomal recessive traits in cats: *Dense* coloration^54^, *Long* hair^58,59^ and points allele (*c*^*s*^) for the *Color* locus^55,56^ and one sex-linked trait, the *Orange* coloration locus^43,60,91,92^, were analyzed. The number of cats used in each analysis is listed in Table 2. For the recessive traits, the case-control associations (*−assoc*) were performed with PLINK^88^ using subsets of samples from the available 2,078 sample dataset. The GWAS to localize *Dense* was performed on three different datasets: random bred cats, Burmese and Birman. Haplotypes for the locus were identified by exporting genotypes from position 216 Mb to position 221 Mb of chromosome C1 and analyzed visually. Haplotypes were exported for each trait using PLINK^88^ 5 Mb 5′ and 3′ of each causal variant and visually inspected. Haplotypes for *Dense* were exported in Chartreaux, Korat and Russian Blue, haplotypes for *Color* were exported in Birman, Burmese and Siamese and haplotypes for *Long* fur were exported for Maine Coon, Norwegian Forest cat, Persian, Ragdoll, Siberian and Turkish Angora in the sample sets used for the GWAS associations or on the available cats in the dataset.

For the X-linked *Orange* association, samples from different breeds were selected. Two analyses were conducted for the cross-breed *Orange* association. The first analysis was performed using chi-square tests for allelic association with individuals from different breeds (–*assoc*) and the second analysis accounts for population stratification by applying the Cochran-Mantel-Haenszel (CMH) test (–*mh*). Cats were clustered for the CMH test on the basis of the pair-wise population concordance (PPC) test, with a p-value of 0.01 set for merging individuals (–*cluster*, –*ppc* 0.01). Only samples and markers with a genotyping rate >90% and markers with MAF ≥ 0.05 were selected for each association analysis independently. Genomic inflation in the association measures of the p-values was evaluated by calculating the genomic inflation factor (ƛ) using PLINK^88^ (–*adjust*). To determine significance, multiple testing correction was accomplished with 100,000 permutation using PLINK^88^ (–*mperm*). T-max permuted p-values were considered genome-wide significant at p < 0.05. A Manhattan plot of the genome-wide p-values and permuted p-values were generated using a custom R script. The haplotype for the *Orange* locus was explored by exporting genotypes from position 105 Mb to positon 109 Mb of the X chromosome and then analyzed visually.

Considering the presence of the causative markers of the three phenotypes on the array, the power of association using the array was calculated by measuring the LD (squared correlation coefficient **–** *r*^2^) between the causative variants and nearby SNPs. The LD between the closest SNP and the causative was used to calculate the power of the current array to detect SNPs density and the sample size needed to detect a significant association^93^.

## Electronic supplementary material

Supplementary information

Supplementary dataset 1

Supplementary dataset 2

Supplementary dataset 3

Supplementary dataset 4

Supplementary dataset 5

Supplementary dataset 6

Supplementary dataset 7

Supplementary dataset 8

Supplementary dataset 9
