# Author Correction: Applications and efficiencies of the first cat 63K DNA array

**DOI:** 10.1038/s41598-018-26885-5

**Published:** 2018-06-04

**Authors:** Barbara Gandolfi, Hasan Alhaddad, Mona Abdi, Leslie H. Bach, Erica K. Creighton, Brian W. Davis, Jared E. Decker, Nicholas H. Dodman, Edward I. Ginns, Jennifer C. Grahn, Robert A. Grahn, Bianca Haase, Jens Haggstrom, Michael J. Hamilton, Christopher R. Helps, Jennifer D. Kurushima, Hannes Lohi, Maria Longeri, Richard Malik, Kathryn M. Meurs, Michael J. Montague, James C. Mullikin, William J. Murphy, Sara M. Nilson, Niels C. Pedersen, Carlyn B. Peterson, Clare Rusbridge, Rashid Saif, G. Diane Shelton, Wesley C. Warren, Muhammad Wasim, Leslie A. Lyons

**Affiliations:** 1 Department of Veterinary Medicine and Surgery, College of Veterinary Medicine, University of Missouri - Columbia, Columbia, MO USA; 2 Department of Biological Sciences, Kuwait University, Safat, Kuwait; 3 Department of Population Health and Reproduction, School of Veterinary Medicine, University of California – Davis, Davis, CA USA; 4 University of San Francisco, San Francisco, CA USA; 5 Department of Veterinary Integrative Biosciences, Texas A&M University, College Station, TX USA; 6 Division of Animal Sciences, University of Missouri - Columbia, Columbia, MO USA; 7 Cummings School of Veterinary Medicine, Tufts University, North Grafton, MA USA; 8 Department of Psychiatry, University of Massachusetts Medical School, Worcester, MA USA; 9 Veterinary Genetics Laboratory, School of Veterinary Medicine, University of California - Davis, Davis, CA USA; 10 Sydney School of Veterinary Science, University of Sydney, Sydney, Australia; 11 Department of Clinical Sciences, Swedish University of Agricultural Sciences, Uppsala, Sweden; 12 Department of Biochemistry, University of California – Riverside, Riverside, CA USA; 13 Langford Vets, University of Bristol, Bristol, United Kingdom; 14 Foothill College, Los Altos Hills, CA USA; 15 Department of Veterinary Biosciences, Research Programs Unit, Molecular Neurology, University of Helsinki, and The Folkhälsan Institute of Genetics, Helsinki, Finland; 16 Department of Veterinary Medicine, Università degli Studi di Milano, Milan, Italy; 17 Centre for Veterinary Education, University of Sydney, New South Wales, Australia; 18 Department of Clinical Sciences, College of Veterinary Medicine, North Carolina State University, Raleigh, NC USA; 19 Department of Neuroscience, Parelman School of Medicine, University of Pennsylvania, Philadelphia, PA USA; 20 NIH Intramural Sequencing Center, National Human Genome Research Institute, National Institutes of Health, Bethesda, MD USA; 21 Center for Companion Animal Health, School of Veterinary Medicine, University of California - Davis, Davis, CA USA; 22 School of Veterinary Medicine, Faculty of Health and Medical Sciences, University of Surrey, Guildford, Surrey, United Kingdom; 23 Institute of Biotechnology, Gulab Devi Educational Complex, Lahore, Pakistan; 24 Department of Pathology, University of California, San Diego, La Jolla, CA USA; 25 McDonnell Genome Institute, Washington University School of Medicine, St Louis, MO USA; 26 Institute of Biochemistry and Biotechnology, University of Veterinary and Animal Sciences, Lahore, Pakistan

Correction to: *Scientific Reports* 10.1038/s41598-018-25438-0, published online 4 May 2018

The original version of this Article contained a typographical error in the spelling of the author G. Diane Shelton, which was incorrectly given as Diane G. Shelton. This has now been corrected in the PDF and HTML versions of the Article and Supplementary Information file.

