# Supplementary material for: Applications and efficiencies of the first cat 63K DNA array
Source: Sci Rep. 2018 May 4;8:7024. doi: 10.1038/s41598-018-25438-0 (PMC5935720; doi:10.1038/s41598-018-25438-0)
Supplement: Supplementary file 1 — Supplementary information [file 41598_2018_25438_MOESM1_ESM.doc]

**Supplementary Files**

**Applications and efficiencies of the first cat 63K DNA array**

Barbara Gandolfi, Hasan Alhaddad, Mona Abdi, Leslie H. Bach, Erica K. Creighton, Brian W. Davis, Jared E. Decker, Nicholas H. Dodman, Edward I. Ginns, Jennifer C. Grahn, Robert A. Grahn, Bianca Haase, Jens Haggstrom, Michael J. Hamilton, Christopher R. Helps, Jennifer D. Kurushima, Hannes Lohi, Maria Longeri, Richard Malik, Kathryn M. Meurs, Michael J. Montague, James C. Mullikin, William J. Murphy, Sara M. Nilson, Niels C. Pedersen, Carlyn B. Peterson, Clare Rusbridge, Rashid Saif, G. Diane Shelton, Wesley C. Warren, Muhammad Wasim, Leslie A. Lyons

**Supplementary Figures**


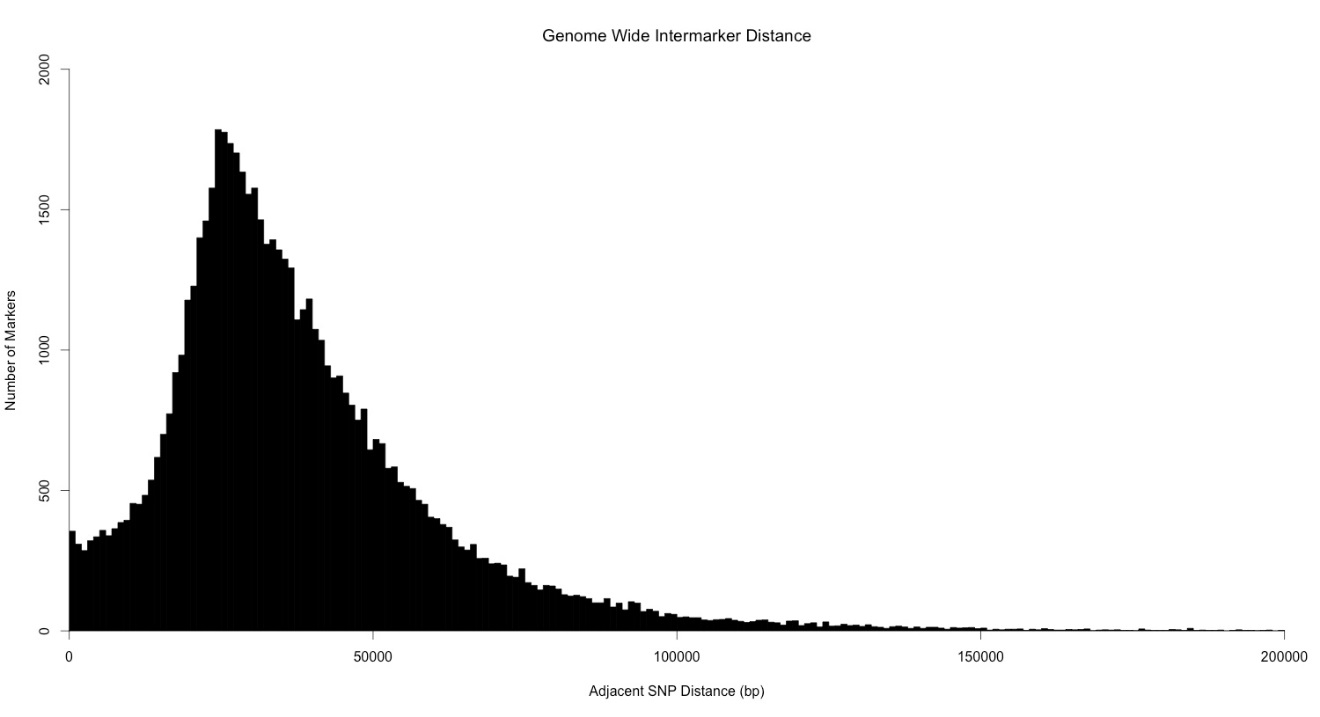


**Supplementary Figure 1. Genome-wide distribution of intermarker distances.** The genome-wide intermarkerdistribution of array loci is plotted as the marker frequency relative to the distance to the closest adjacent array SNP.


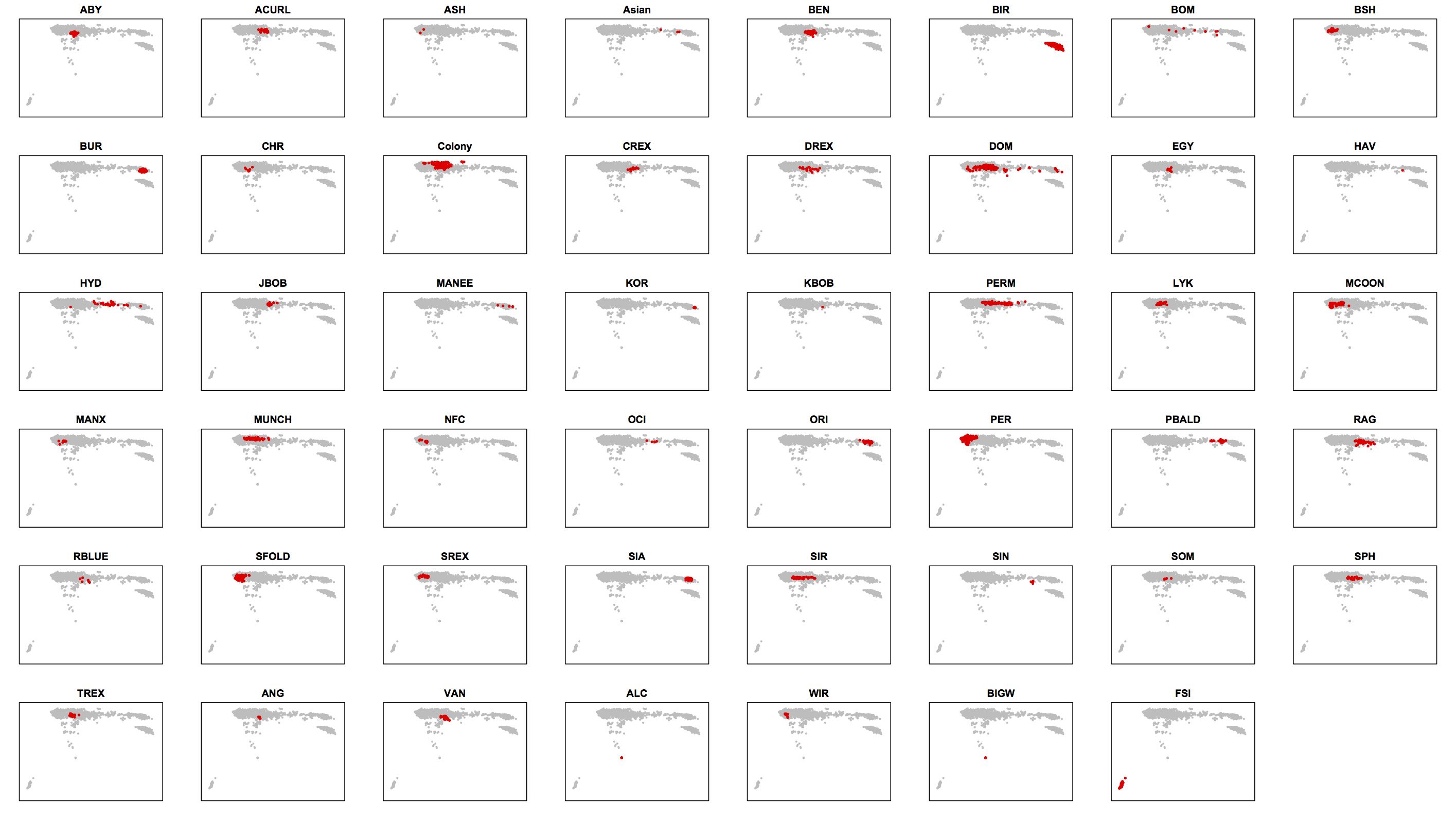


a.


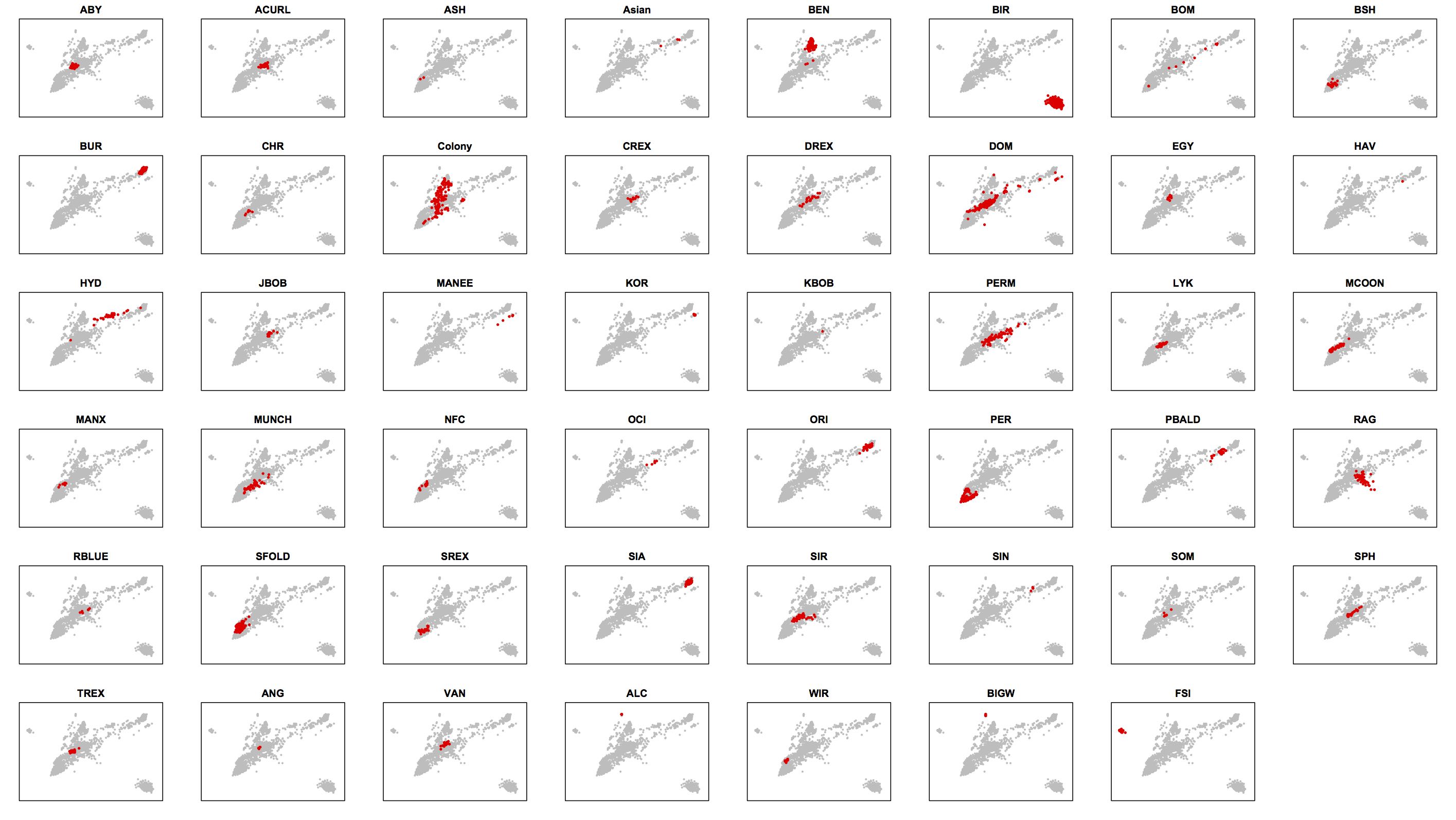


b.


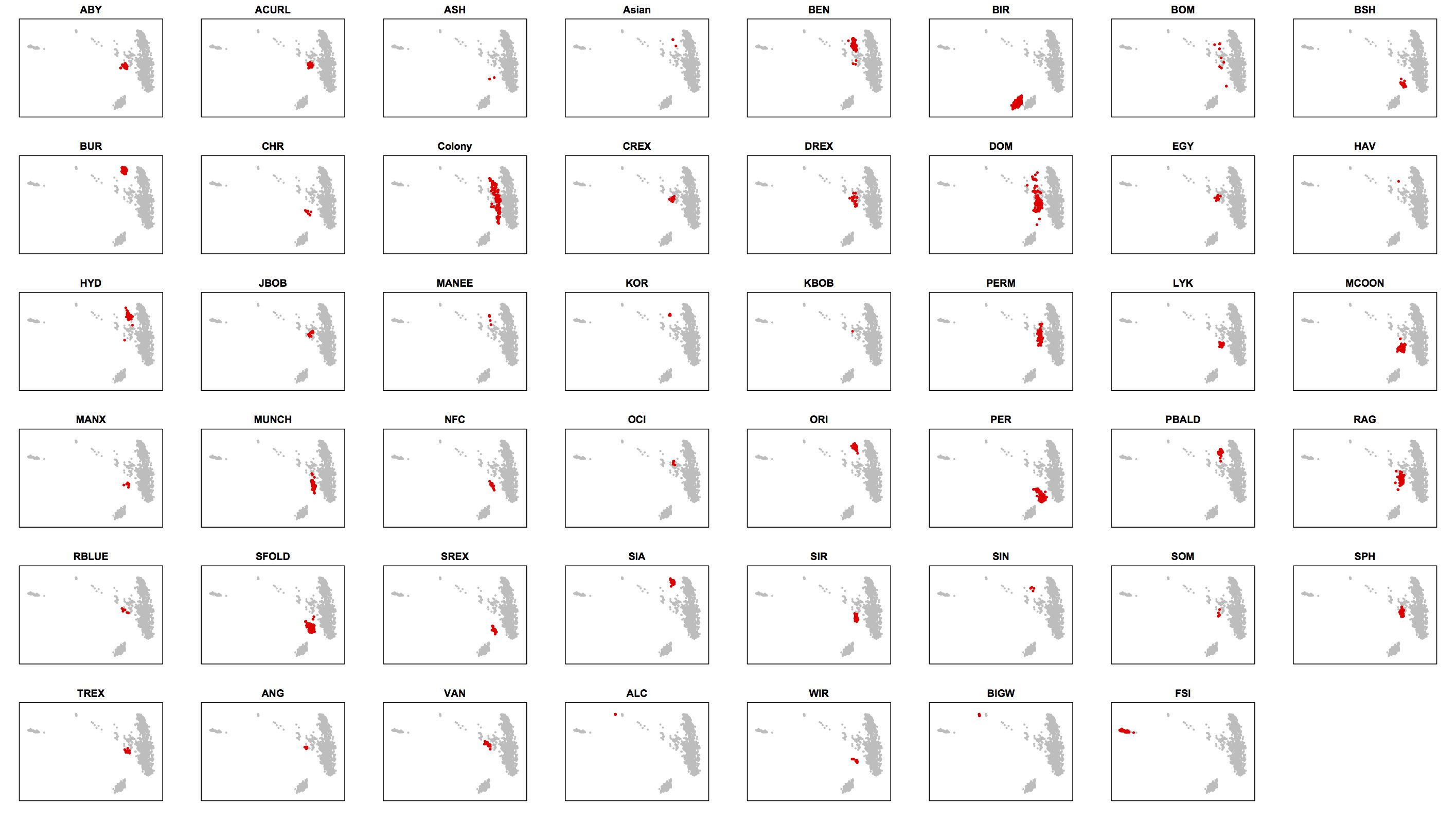


c.

**Supplementary Figure 2: Detailed genetic structure of cat populations using Feline array markers.** Gray dots represent the individual cats position in the two dimensions plotted while red dots represent the individuals that belong to a specific population. An abbreviated name of population under investigation (red dots) is used as a header for the plot and details can be found in table#. (a-c) are plots of dimension1 vs. dimension2, dimension1 vs. dimension3, dimension2 vs. dimension3, respectively.


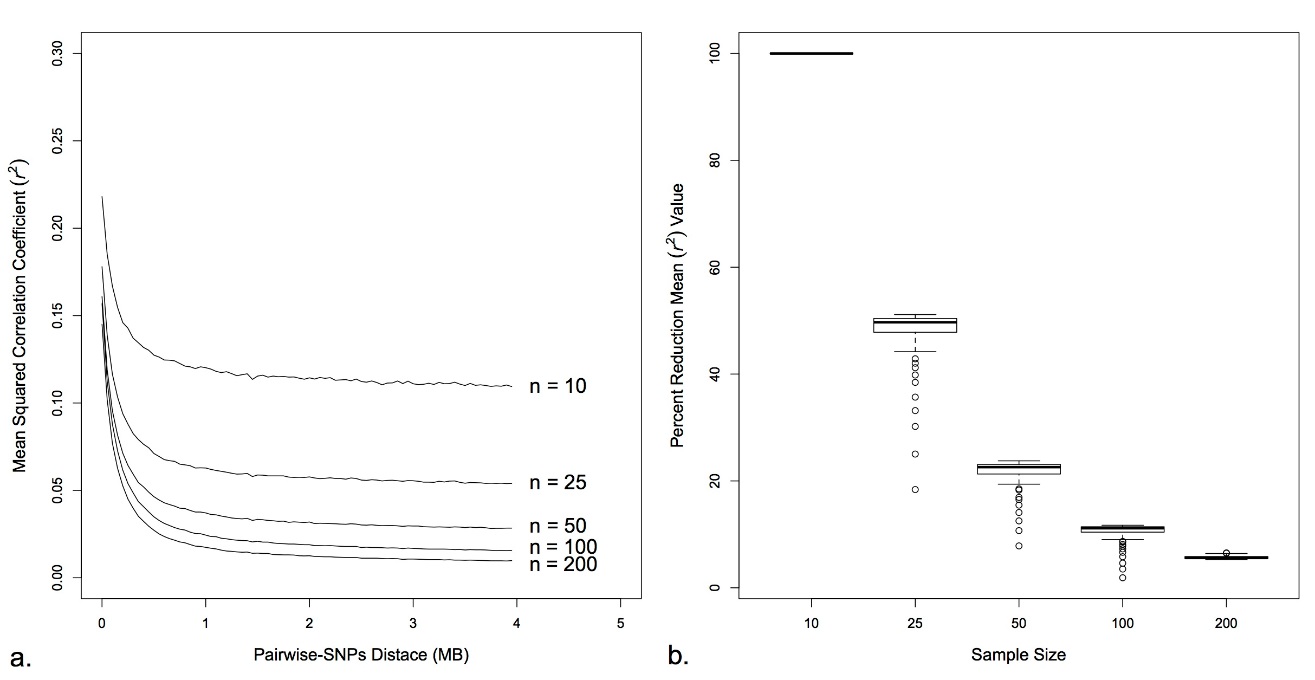


**Supplementary Figure 3. Effects of sample size on linkage disequilibrium estimates.** (a) Decay of linkage disequilibrium as a function of distance (Mb) for different sample size (n = 10, 25, 50, 100, 200) of a random bred population. (b) The reduction in the linkage disequilibrium estimates as the sample size increases. Increasing sample size from 10 to 25 reduces estimates by ~ 50% (details of the calculations are in Materials and Methods).

**
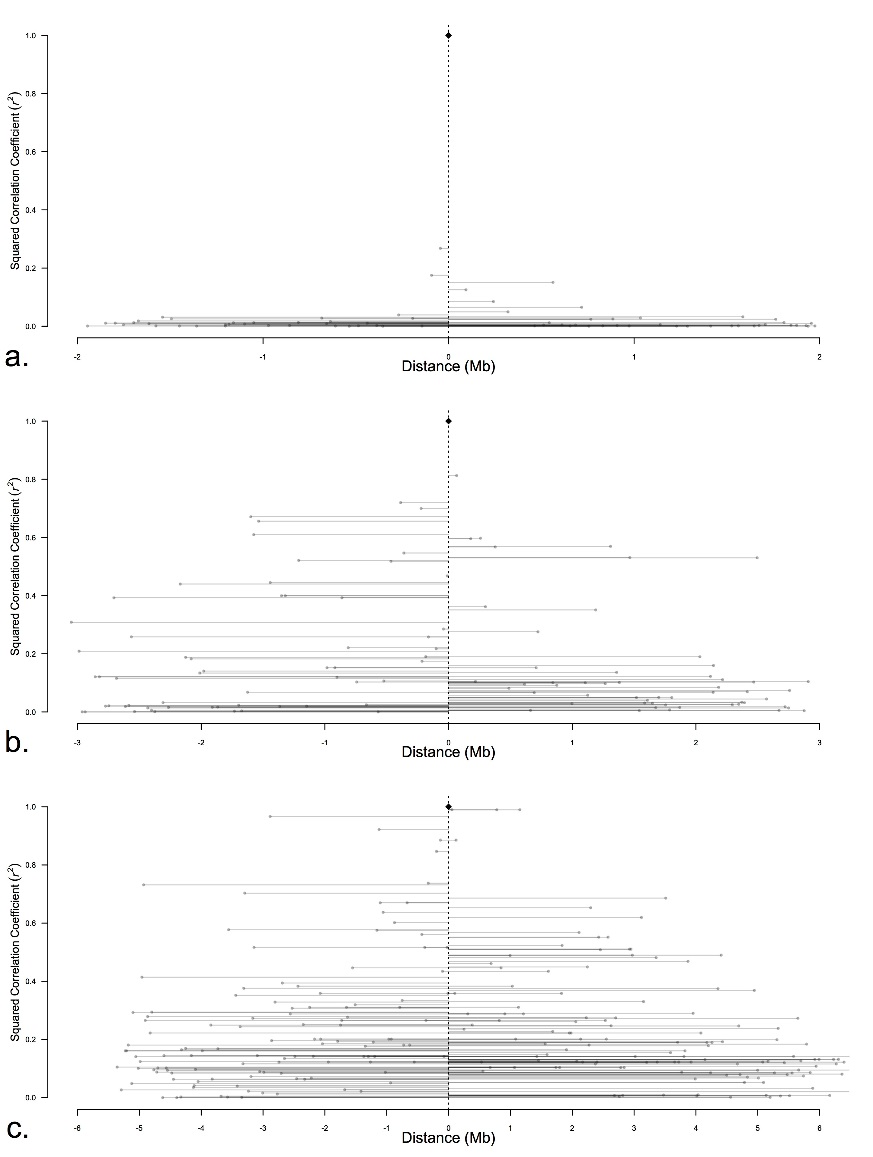
**

**Supplementary Figure 4. Linkage disequilibrium between causative mutations and surrounding markers.** a-c) vertical dashed line and black dot represent the causative marker of dilute coloration, long hair, and point coloration, respectively. Gray dots represent the SNPs and horizontal lines correspond to the distance to the causative marker. The y-axis is a measure of pair-wise linkage disequilibrium (r2) between the causative markers and nearby SNPs.

**
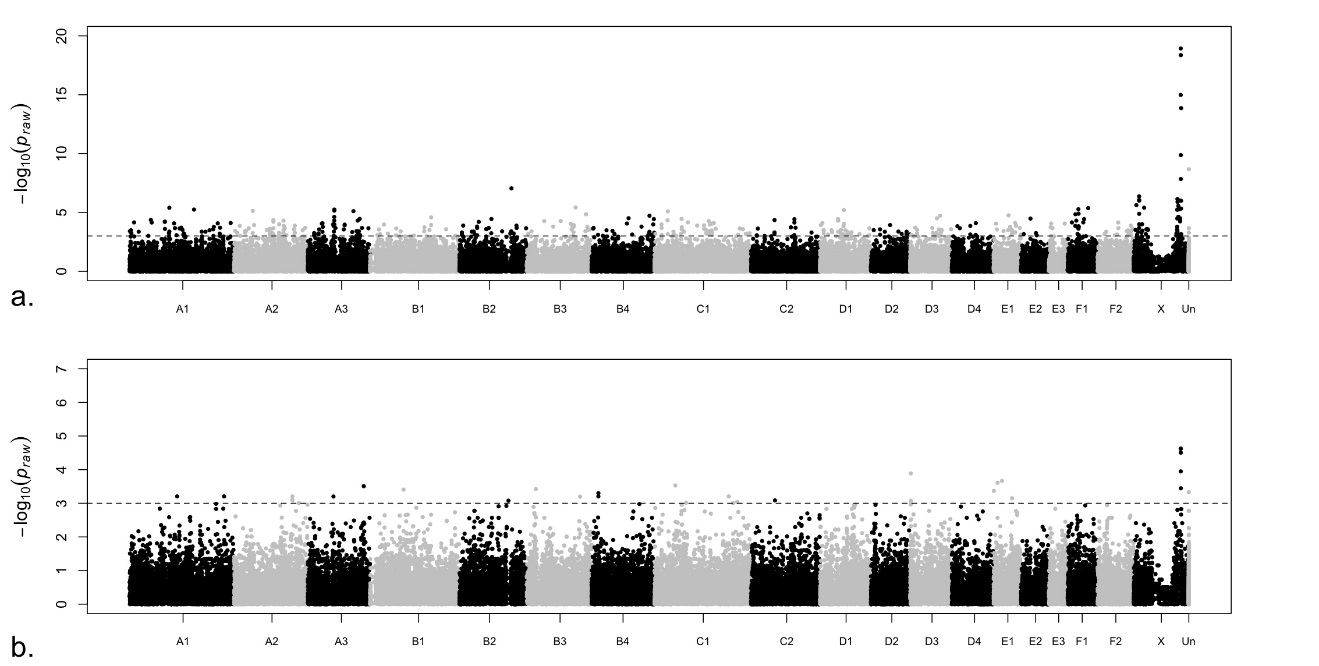
**

**Supplementary Figure 5. Genome-wide association analysis of orange coloration in cats.** a) Manhattan plot of the allelic association study across multiple breeds b) CMH association study using the same dataset of the allelic association after pair-wise population concordance (PPC) test.


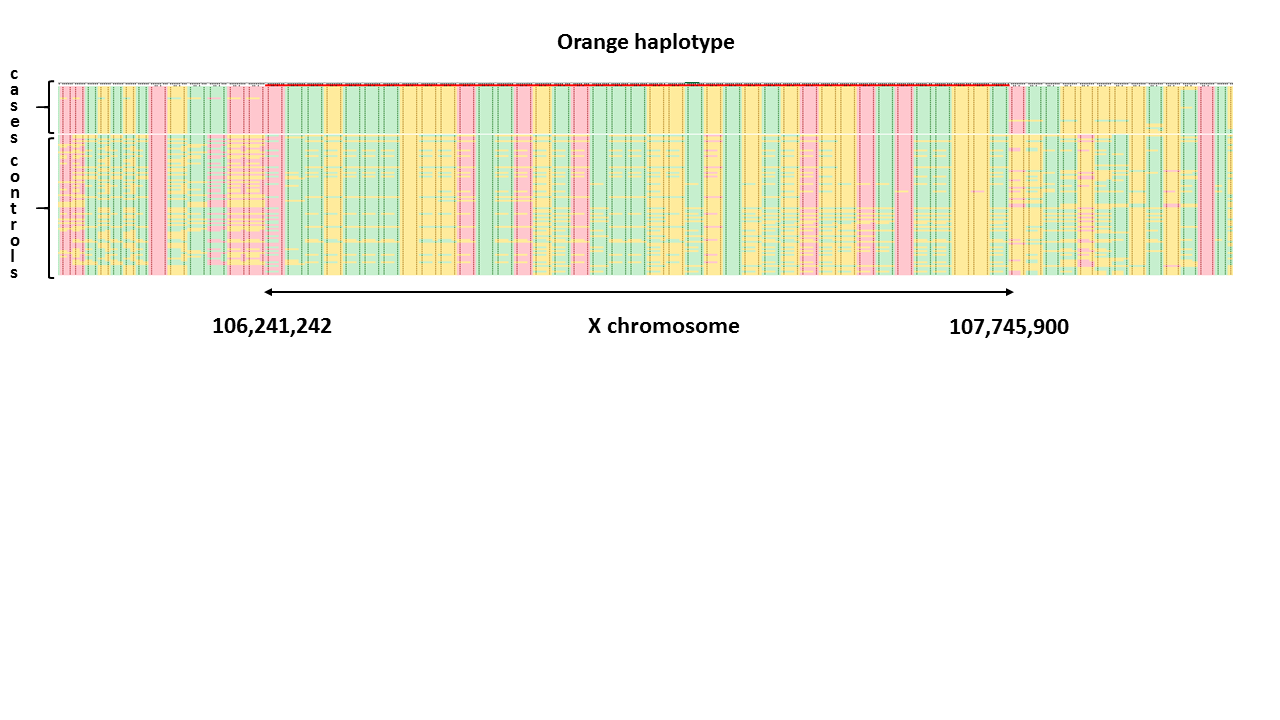


**Supplementary Figure 6**. Extent of the orange coloration haplotype. A haplotype block was identified between al cases from position 106,241,242 to position 107,745,900 of the X chromosome.

**Supplementary Tables**

**Supplementary Table 1**. Number of markers and average distance for each chromosome.

| Chromosome | # of markers | Average distance (bp) |
| --- | --- | --- |
| A1 | 6369 | 37741 |
| A2 | 4424 | 38113 |
| A3 | 3444 | 40867 |
| B1 | 5086 | 40569 |
| B2 | 3685 | 41523 |
| B3 | 3746 | 39488 |
| B4 | 3760 | 37870 |
| C1 | 5835 | 38081 |
| C2 | 4283 | 37181 |
| D1 | 3109 | 37048 |
| D2 | 2367 | 37184 |
| D3 | 2555 | 36699 |
| D4 | 2491 | 37893 |
| E1 | 1564 | 38961 |
| E2 | 1679 | 36784 |
| E3 | 1104 | 37122 |
| F1 | 1799 | 38963 |
| F2 | 2169 | 38701 |
| X | 2724 | 46697 |

**Supplementary Table 2. Genotypes mismatches between duplicated samples.** Duplicated samples mismatches number and % including the whole SNPs dataset, and after excluding SNPs with a genotyping rate below 10% and with Mendelian errors.

|  |  | 62897 | | 62272 | | 62051 | |
| --- | --- | --- | --- | --- | --- | --- | --- |
| Sample ID | Category | # mismatch | % mismatch | # mismatch | % mismatch | # mismatch | % mismatch |
| CCL94 | control | 356 | 0.57 | 344 | 0.55 | 340 | 0.55 |
| Cinnamon | control | 0 | 0 | 0 | 0 | 0 | 0 |
| 4406 | control | 4 | 0.006 | 3 | 0.005 | 3 | 0.005 |
| 4649 | control | 4 | 0.006 | 3 | 0.005 | 3 | 0.005 |
| Cinnamon vs wga12682 | WGA test | 1720 | 2.73 | 1639 | 2.63 | 1628 | 2.62 |
| 16216T | tumor | 439 | 0.7 | 429 | 0.69 | 426 | 0.69 |
| 6223T | tumor | 681 | 1.08 | 664 | 1.07 | 660 | 1.06 |
| 10208 | Involuntary | 223 | 0.35 | 170 | 0.27 | 164 | 0.26 |
| 10209 | Involuntary | 78 | 0.12 | 46 | 0.07 | 44 | 0.07 |
| 10850 | Involuntary | 331 | 0.52 | 299 | 0.48 | 297 | 0.48 |
| 13106 | Involuntary | 353 | 0.56 | 320 | 0.51 | 318 | 0.51 |
| 13226 | Involuntary | 648 | 1.03 | 492 | 0.79 | 456 | 0.73 |
| 13233 | Involuntary | 446 | 0.71 | 310 | 0.5 | 275 | 0.44 |
| 15427 | Involuntary | 702 | 1.11 | 567 | 0.91 | 525 | 0.85 |
| 17993 | Involuntary | 462 | 0.73 | 353 | 0.57 | 331 | 0.53 |
| 5588 | Involuntary | 541 | 0.86 | 410 | 0.66 | 383 | 0.62 |
| 7359 | Involuntary | 550 | 0.87 | 497 | 0.8 | 491 | 0.8 |
| 8638 | Involuntary | 530 | 0.84 | 441 | 0.71 | 431 | 0.7 |
| 9793 | Involuntary | 279 | 0.44 | 225 | 0.36 | 213 | 0.34 |
| LYM16513 | Involuntary | 443 | 0.7 | 426 | 0.68 | 426 | 0.68 |
| 1310 | Low calls | 28056 | 44.6 | 27749 | 44.56 | 27652 | 44.56 |
| 13632 | Low calls | 5810 | 9.23 | 5600 | 9 | 5551 | 8.9 |
| 15723 | Low calls | 23959 | 38.1 | 23692 | 38.05 | 23589 | 38.01 |
| 4439 | Low calls | 29346 | 46.7 | 28968 | 46.5 | 28848 | 46.5 |
| 5589 | Low calls | 27338 | 43.5 | 26976 | 43.3 | 26861 | 43.3 |
| 6667 | Low calls | 3952 | 6.3 | 3799 | 6.1 | 3756 | 6.05 |
| 10988 | Low calls | 2711 | 4.3 | 2548 | 4.09 | 2514 | 4.05 |

**Supplementary Table 3**. Number of SNPs in each MAF ranges.

| MAF bin | Number SNPs | MAF bin | Number SNPs |
| --- | --- | --- | --- |
| 0-0.05 | 7813 | 0-0.005 | 2013 |
| 0.05-0.1 | 7537 | 0.005-0.01 | 615 |
| 0.1-0.15 | 7469 | 0.01-0.015 | 589 |
| 0.15-0.2 | 7187 | 0.015-0.02 | 567 |
| 0.2-0.25 | 6477 | 0.02-0.025 | 626 |
| 0.25-0.3 | 5977 | 0.025-0.03 | 677 |
| 0.3-0.35 | 5327 | 0.03-0.035 | 690 |
| 0.35-0.4 | 5027 | 0.035-0.04 | 683 |
| 0.4-0.45 | 4797 | 0.04-0.045 | 675 |
| 0.45-0.5 | 4661 | 0.045-0.05 | 678 |
|  |  | 0.05-0.055 | 691 |
|  |  | 0.055-0.06 | 756 |
|  |  | 0.06-0.065 | 741 |
|  |  | 0.065-0.07 | 724 |
|  |  | 0.07-0.075 | 756 |
|  |  | 0.075-0.08 | 797 |
|  |  | 0.08-0.085 | 736 |
|  |  | 0.085-0.09 | 787 |
|  |  | 0.09-0.095 | 788 |
|  |  | 0.095-0.1 | 761 |
|  |  | 0.1-0.105 | 756 |
|  |  | 0.105-0.11 | 759 |
|  |  | 0.11-0.115 | 776 |
|  |  | 0.115-0.12 | 698 |
|  |  | 0.12-0.125 | 790 |
|  |  | 0.125-0.13 | 762 |
|  |  | 0.13-0.135 | 748 |
|  |  | 0.135-0.14 | 741 |
|  |  | 0.14-0.145 | 732 |
|  |  | 0.145-0.15 | 707 |
|  |  | 0.15-0.155 | 756 |
|  |  | 0.155-0.16 | 723 |
|  |  | 0.16-0.165 | 753 |
|  |  | 0.165-0.17 | 775 |
|  |  | 0.17-0.175 | 733 |
|  |  | 0.175-0.18 | 661 |
|  |  | 0.18-0.185 | 699 |
|  |  | 0.185-0.19 | 670 |
|  |  | 0.19-0.195 | 689 |
|  |  | 0.195-0.2 | 728 |
|  |  | 0.2-0.205 | 676 |
|  |  | 0.205-0.21 | 695 |
|  |  | 0.21-0.215 | 673 |
|  |  | 0.215-0.22 | 635 |
|  |  | 0.22-0.225 | 623 |
|  |  | 0.225-0.23 | 607 |
|  |  | 0.23-0.235 | 639 |
|  |  | 0.235-0.24 | 625 |
|  |  | 0.24-0.245 | 669 |
|  |  | 0.245-0.25 | 635 |
|  |  | 0.25-0.255 | 625 |
|  |  | 0.255-0.26 | 653 |
|  |  | 0.26-0.265 | 654 |
|  |  | 0.265-0.27 | 553 |
|  |  | 0.27-0.275 | 590 |
|  |  | 0.275-0.28 | 603 |
|  |  | 0.28-0.285 | 609 |
|  |  | 0.285-0.29 | 576 |
|  |  | 0.29-0.295 | 543 |
|  |  | 0.295-0.3 | 571 |
|  |  | 0.3-0.305 | 565 |
|  |  | 0.305-0.31 | 519 |
|  |  | 0.31-0.315 | 534 |
|  |  | 0.315-0.32 | 547 |
|  |  | 0.32-0.325 | 553 |
|  |  | 0.325-0.33 | 523 |
|  |  | 0.33-0.335 | 515 |
|  |  | 0.335-0.34 | 529 |
|  |  | 0.34-0.345 | 533 |
|  |  | 0.345-0.35 | 509 |
|  |  | 0.35-0.355 | 536 |
|  |  | 0.355-0.36 | 492 |
|  |  | 0.36-0.365 | 500 |
|  |  | 0.365-0.37 | 491 |
|  |  | 0.37-0.375 | 503 |
|  |  | 0.375-0.38 | 491 |
|  |  | 0.38-0.385 | 496 |
|  |  | 0.385-0.39 | 523 |
|  |  | 0.39-0.395 | 492 |
|  |  | 0.395-0.4 | 503 |
|  |  | 0.4-0.405 | 491 |
|  |  | 0.405-0.41 | 428 |
|  |  | 0.41-0.415 | 492 |
|  |  | 0.415-0.42 | 439 |
|  |  | 0.42-0.425 | 460 |
|  |  | 0.425-0.43 | 483 |
|  |  | 0.43-0.435 | 527 |
|  |  | 0.435-0.44 | 500 |
|  |  | 0.44-0.445 | 508 |
|  |  | 0.445-0.45 | 469 |
|  |  | 0.45-0.455 | 477 |
|  |  | 0.455-0.46 | 509 |
|  |  | 0.46-0.465 | 426 |
|  |  | 0.465-0.47 | 442 |
|  |  | 0.47-0.475 | 465 |
|  |  | 0.475-0.48 | 463 |
|  |  | 0.48-0.485 | 477 |
|  |  | 0.485-0.49 | 477 |
|  |  | 0.49-0.495 | 466 |
|  |  | 0.495-0.5 | 459 |

**Supplementary Table 4**. Effect of sample size of random bred cats on average squared correlation coefficient (*r^2^*) estimates of LD at different distance bins.

| **Distance** | **DOM10** | **DOM25** | **DOM50** | **DOM100** | **DOM200** |
| --- | --- | --- | --- | --- | --- |
| **0** | 0.2181 | 0.178 | 0.1609 | 0.1568 | 0.1451 |
| **50000** | 0.185 | 0.1387 | 0.1189 | 0.1124 | 0.1018 |
| **100000** | 0.1669 | 0.1165 | 0.0956 | 0.0879 | 0.077 |
| **150000** | 0.1547 | 0.1034 | 0.0816 | 0.0726 | 0.0626 |
| **200000** | 0.146 | 0.0939 | 0.0713 | 0.0616 | 0.0526 |
| **250000** | 0.1428 | 0.0879 | 0.0643 | 0.0539 | 0.045 |
| **300000** | 0.1371 | 0.0825 | 0.0593 | 0.0486 | 0.0398 |
| **350000** | 0.1345 | 0.0791 | 0.0545 | 0.0436 | 0.0352 |
| **400000** | 0.1318 | 0.0764 | 0.052 | 0.0407 | 0.0323 |
| **450000** | 0.1302 | 0.0744 | 0.049 | 0.0377 | 0.0295 |
| **500000** | 0.1274 | 0.0711 | 0.0464 | 0.0349 | 0.0272 |
| **550000** | 0.1263 | 0.0694 | 0.0445 | 0.0328 | 0.0251 |
| **600000** | 0.1246 | 0.0676 | 0.0429 | 0.0311 | 0.0236 |
| **650000** | 0.1245 | 0.067 | 0.0419 | 0.0299 | 0.0224 |
| **700000** | 0.1241 | 0.0666 | 0.041 | 0.0287 | 0.0215 |
| **750000** | 0.1226 | 0.065 | 0.0398 | 0.0278 | 0.0205 |
| **800000** | 0.1211 | 0.0646 | 0.0396 | 0.0273 | 0.02 |
| **850000** | 0.1207 | 0.0641 | 0.0385 | 0.0261 | 0.019 |
| **900000** | 0.1197 | 0.0628 | 0.0376 | 0.0253 | 0.0181 |
| **950000** | 0.1207 | 0.0629 | 0.0376 | 0.0252 | 0.0179 |
| **1000000** | 0.1202 | 0.0628 | 0.0371 | 0.0244 | 0.0174 |
| **1050000** | 0.1194 | 0.062 | 0.0362 | 0.0236 | 0.0169 |
| **1100000** | 0.1181 | 0.0614 | 0.036 | 0.0235 | 0.0165 |
| **1150000** | 0.1174 | 0.0609 | 0.0353 | 0.0226 | 0.0158 |
| **1200000** | 0.1179 | 0.0604 | 0.0348 | 0.0223 | 0.0153 |
| **1250000** | 0.1168 | 0.0598 | 0.0342 | 0.0218 | 0.0151 |
| **1300000** | 0.1156 | 0.0593 | 0.0339 | 0.0215 | 0.0148 |
| **1350000** | 0.1162 | 0.0594 | 0.0337 | 0.0213 | 0.0146 |
| **1400000** | 0.1167 | 0.0597 | 0.034 | 0.0212 | 0.0147 |
| **1450000** | 0.1135 | 0.0579 | 0.0328 | 0.0205 | 0.014 |
| **1500000** | 0.1154 | 0.0588 | 0.0334 | 0.0209 | 0.0141 |
| **1550000** | 0.1159 | 0.0587 | 0.0332 | 0.0204 | 0.0139 |
| **1600000** | 0.1148 | 0.0583 | 0.0328 | 0.0204 | 0.0139 |
| **1650000** | 0.1154 | 0.0583 | 0.0326 | 0.0199 | 0.0133 |
| **1700000** | 0.115 | 0.0583 | 0.0323 | 0.0196 | 0.0132 |
| **1750000** | 0.1152 | 0.0583 | 0.0324 | 0.0195 | 0.0131 |
| **1800000** | 0.1148 | 0.0576 | 0.0317 | 0.0193 | 0.0131 |
| **1850000** | 0.1149 | 0.0574 | 0.032 | 0.0193 | 0.013 |
| **1900000** | 0.1142 | 0.0575 | 0.0318 | 0.0191 | 0.0126 |
| **1950000** | 0.1136 | 0.0574 | 0.0316 | 0.0189 | 0.0125 |
| **2000000** | 0.1144 | 0.0577 | 0.0319 | 0.0189 | 0.0126 |
| **2050000** | 0.1137 | 0.0571 | 0.031 | 0.0184 | 0.0122 |
| **2100000** | 0.1146 | 0.0567 | 0.0312 | 0.0184 | 0.0121 |
| **2150000** | 0.1141 | 0.0569 | 0.0311 | 0.0183 | 0.012 |
| **2200000** | 0.1145 | 0.0572 | 0.031 | 0.0184 | 0.012 |
| **2250000** | 0.113 | 0.0568 | 0.0309 | 0.0182 | 0.0118 |
| **2300000** | 0.1132 | 0.0568 | 0.0307 | 0.018 | 0.0117 |
| **2350000** | 0.1134 | 0.0564 | 0.0306 | 0.0179 | 0.0116 |
| **2400000** | 0.1127 | 0.0571 | 0.0309 | 0.0179 | 0.0116 |
| **2450000** | 0.1137 | 0.0568 | 0.0307 | 0.0179 | 0.0116 |
| **2500000** | 0.1123 | 0.0559 | 0.0302 | 0.0173 | 0.0112 |
| **2550000** | 0.1119 | 0.0557 | 0.0301 | 0.0175 | 0.0112 |
| **2600000** | 0.1126 | 0.0561 | 0.0303 | 0.0173 | 0.0112 |
| **2650000** | 0.1117 | 0.0559 | 0.03 | 0.0173 | 0.0112 |
| **2700000** | 0.1104 | 0.0554 | 0.0298 | 0.0172 | 0.011 |
| **2750000** | 0.1114 | 0.0557 | 0.0299 | 0.017 | 0.0109 |
| **2800000** | 0.1115 | 0.0558 | 0.0299 | 0.0171 | 0.011 |
| **2850000** | 0.1126 | 0.0555 | 0.0297 | 0.017 | 0.0109 |
| **2900000** | 0.1111 | 0.0552 | 0.0294 | 0.0167 | 0.0105 |
| **2950000** | 0.1125 | 0.0556 | 0.0297 | 0.017 | 0.0107 |
| **3000000** | 0.111 | 0.0555 | 0.0296 | 0.0168 | 0.0107 |
| **3050000** | 0.1105 | 0.0552 | 0.0296 | 0.0167 | 0.0106 |
| **3100000** | 0.1107 | 0.0547 | 0.0292 | 0.0165 | 0.0105 |
| **3150000** | 0.1113 | 0.0547 | 0.0291 | 0.0164 | 0.0104 |
| **3200000** | 0.1106 | 0.0546 | 0.029 | 0.0164 | 0.0104 |
| **3250000** | 0.1118 | 0.0552 | 0.0291 | 0.0164 | 0.0104 |
| **3300000** | 0.111 | 0.0548 | 0.029 | 0.0164 | 0.0102 |
| **3350000** | 0.1111 | 0.0553 | 0.0289 | 0.0163 | 0.0103 |
| **3400000** | 0.1119 | 0.0554 | 0.0291 | 0.0161 | 0.0101 |
| **3450000** | 0.1108 | 0.0546 | 0.029 | 0.0162 | 0.0101 |
| **3500000** | 0.11 | 0.0541 | 0.0287 | 0.0159 | 0.01 |
| **3550000** | 0.1112 | 0.0545 | 0.029 | 0.0161 | 0.0101 |
| **3600000** | 0.1101 | 0.0544 | 0.0287 | 0.016 | 0.01 |
| **3650000** | 0.1104 | 0.0543 | 0.0289 | 0.016 | 0.0099 |
| **3700000** | 0.11 | 0.054 | 0.0286 | 0.0159 | 0.0098 |
| **3750000** | 0.1095 | 0.0541 | 0.0282 | 0.0157 | 0.0097 |
| **3800000** | 0.1098 | 0.054 | 0.0283 | 0.0155 | 0.0097 |
| **3850000** | 0.1096 | 0.0541 | 0.0283 | 0.0156 | 0.0097 |
| **3900000** | 0.1103 | 0.0539 | 0.0284 | 0.0155 | 0.0096 |
| **3950000** | 0.1094 | 0.054 | 0.0284 | 0.0156 | 0.0098 |
| **4000000** | 0.0565 | 0.031 | 0.0535 | 0.019 | 0.004 |
| **5000000** | 0.109 | 0.0534 | 0.0278 | 0.0151 | 0.0093 |
| **6000000** | 0.0992 | 0.0462 | 0.0226 | 0.0112 | 0.0062 |

**Supplementary Table 5**. Details of GWAS association results.

| Trait | Association | Chro | SNPs | Raw *P_values_* | *P_genome_*  values |
| --- | --- | --- | --- | --- | --- |
| Dilution | Allelic | C1 | 218,100,114 (within *MLPH*) | 1.3e-20 | 0.00002 |
|  |  | C1 | 218,055,908 | 2.1e-5 | 0.8278 |
|  |  | E1 | 61,374,140 | 2.2e-5 | 0.8548 |
| Long hair | Allelic | B1 | 140,077,554 (within *FGF5*) | 8.2e-10 | 0.0001 |
|  |  | B1 | 140,143,322 | 9.5e-8 | 0.0065 |
|  |  | B1 | 138,478,128 | 1.9e-6 | 0.07582 |
| Point coloration | Allelic | D1 | 46,341,460 | 2e-9 | 0.00036 |
|  |  | D1 | 46,396,786 (within *TYR*) | 2.2e-9 | 0.00044 |
|  |  | D1 | 47,121,808 | 2.2e-9 | 0.00044 |
| Orange | Allelic | X | 107,777,134 | 1.8e-19 | 0.00002 |
|  |  | X | 107,994,240 | 4.3e-19 | 0.00002 |
|  |  | X | 107,822,242 | 1e-15 | 0.00002 |
| Orange | CMH | X | 107,777,134 | 4.4e-5 | 0.04776 |
|  |  | X | 107,822,242 | 4.4e-5 | 0.04776 |
|  |  | X | 107,994,240 | 3.1e-5 | 0.0751 |

**Supplementary Table 6**. List of genes within the Orange haplotype on the X chromosome.

| **Gene ID** | **Gene name** |
| --- | --- |
| *ZNF280D* | *Zinc finger protein 280C isoform 2* |
| *TSPAN13* | *Tetraspanin-13* |
| *SLC25A14* | *Brain mitochondrial carrier protein 1 isoform 1 precursor* |
| *GPR119* | *Glucose-dependent insulinotropic receptor* |
| *RBMX2* | *RNA-binding motif protein X-linked 2* |
| *FSIP2* | *Fibrous sheath-interacting protein 2* |
| *ENOX2* | *Ecto-NOX disulfide-thiol exchanger 2 isoform b* |
| *LINC01201* | *LINC01201* |
| *ARHGAP36* | *Rho GTPase-activating protein 36 isoform 1 precursor* |
| *IGSF1* | *Immunoglobulin superfamily member 1 isoform 4* |
| *RPL13P5* | *RPL13P5* |
| *FIRRE* | *FIRRE* |

**Supplementary Data Files**

**Supplementary Data File 1.** List of wildcat SNPs

**Supplementary Data File 2.** Map file of the 62,897 SNPs map to *Felis catus* (FelCat 8.0) assembly

**Supplementary Data File 3.** Remapping information of the 62,897 SNPs *to Felis catus* (FelCat8) assembly*,* including position of the SNPs, intermarker distances and gap sizes

**Supplementary Data File 4.** Comparison between the map files generated using *Felis catus* FelCat 6.2 and FelCat 8.0 assemblies

**Supplementary Data File 5.** Genotype dataset of all cats included in the study (ped file)

**Supplementary Data File 6.** List of SNPs with >10% missing data across all samples in the dataset

**Supplementary Data File 7.** List of SNPs with Mendelian errors

**Supplementary Data File 8.** List of hemizogous SNPs

**Supplementary Data File 9.** SNPs MAF for each chromosome
